# Precipitation‐Modulated Harmonic Architectures Enable Superior Strength–Ductility Synergy from Cryogenic to Elevated Temperatures in Nanostructured Alloys

**DOI:** 10.1002/advs.75356

**Published:** 2026-04-23

**Authors:** Wei Li, Liangyin Xiong, Mengchao Niu, Junhua Luan, Wei Wang, Zengbao Jiao

**Affiliations:** ^1^ Institute of Metal Research Chinese Academy of Sciences Shenyang China; ^2^ Department of Mechanical Engineering Research Institute for Advanced Manufacturing the Hong Kong Polytechnic University Hong Kong China; ^3^ School of Materials Science and Engineering University of Science and Technology of China Shenyang China; ^4^ Center For Advanced Nuclear Safety and Sustainable Development Inter‐university 3D Atom Probe Tomography Unit City University of Hong Kong Hong Kong China; ^5^ The Hong Kong Polytechnic University Shenzhen Research Institute Shenzhen China

**Keywords:** deformation microstructure, high entropy alloy, nanoscale precipitation, recrystallization, strengthening mechanism

## Abstract

High‐performance materials that exhibit a robust strength–ductility synergy across cryogenic to high temperatures are essential for aerospace applications, yet achieving this combination remains a significant challenge in materials development. Here, we report that remarkable mechanical properties across a broad temperature range can be achieved in bimodal harmonic‐architectured (BHA) alloys. In this architecture, spherical precipitates stabilize coarse‐grained cores, while lamellar precipitates facilitate selective recrystallization, resulting in a reproducible necklace‐like topology. This engineered microstructure delivers exceptional mechanical performance from −196 °C to 700 °C, consistently achieving yield strengths of 1–2 GPa and ductilities exceeding 10% throughout the entire temperature spectrum. Quantitative analysis reveals that precipitation and grain‐boundary strengthening are the primary contributors to strength at all temperatures, whereas the contribution of dislocation hardening decreases progressively with increasing temperature. The deformation mechanisms exhibit temperature‐adaptive cooperation: dislocation forests and nanotwins enhance deformation at cryogenic temperatures, dislocation–precipitate interactions dominate plasticity at ambient conditions, and interfacial back‐stress accommodation ensures coordinated deformation of bimodal grains at elevated temperatures. This adaptive synergy effectively suppresses both cryogenic embrittlement and high‐temperature softening, establishing a robust structural foundation for broad service applicability. The BHA engineering offers a versatile pathway for developing next‐generation alloys with superior properties required for wide‐temperature applications.

## Graphical abstract

Precipitation‐modulated recrystallization enables a programmable bimodal harmonic architecture in a high‐entropy alloy, delivering 1–2 GPa yield strength with >10% ductility from −196 °C to 700 °C. The resulting broad‐temperature robustness arises from the synergy of dual‐mode nanoprecipitation, harmonic core–shell topology, and temperature‐adaptive deformation mechanisms.

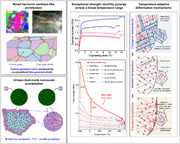

## 1 Introduction

Advanced alloys that can simultaneously sustain high strength and ductility from cryogenic to elevated temperatures are essential for critical applications, such as spacecraft structures, reusable launch vehicles, and rocket propulsion systems. However, most existing alloys exhibit pronounced temperature‐dependent trade‐offs. For instance, face‐centered cubic (FCC) alloys, such as austenitic stainless steels and Cantor alloys, offer excellent ductility at cryogenic temperatures but become relatively soft, with a yield strength of 200 MPa, at temperatures above 500 °C [1, 2]. In contrast, refractory alloys retain their strength at temperatures above 1000 °C but display severely limited plasticity at both room and cryogenic temperatures [3, 4]. Many superalloys achieve exceptional strength at high temperatures but are prone to brittleness at intermediate temperatures (typically 600–800 °C), a phenomenon known as intermediate‐temperature embrittlement [5, 6]. Consequently, designing structural materials with a robust strength–ductility synergy across a broad temperature range remains a significant challenge in materials science.

Recent studies suggest that heterogeneous microstructures provide a promising route toward breaking the strength–ductility trade‐off at certain temperatures [7, 8]. By integrating coarse and fine grains, heterostructures facilitate strain partitioning, interface‐mediated strengthening, and the activation of diverse deformation pathways [9]. These effects significantly enhance work hardening and delay strain localization, resulting in superior strength–ductility combinations in various alloys. However, most reported heterostructures are formed stochastically through dynamic recrystallization or strain‐gradient subdivision, leading to irregular morphologies that are difficult to control. Among various topologies, necklace‐like harmonic grain structures, where coarse grains are encircled by a contiguous network of fine grains, are particularly attractive [10]. This architecture not only enables efficient strain partitioning and dislocation storage but also mitigates the adverse effects of temperature fluctuations, delivering remarkable synergy between strength and ductility across a broad temperature spectrum [11, 12]. Notably, necklace‐like harmonic architectures offer unique advantages under extreme conditions: they enhance cryogenic toughness through twinning‐ and/or TRIP‐assisted plasticity, and improve high‐temperature ductility by accommodating grain boundary sliding along fine‐grained shells. Recent multiscale simulations further confirm that an interconnected shell network provides more uniform stress distribution and suppresses premature localization compared to random bimodal morphologies. These advances highlight necklace‐like harmonic architectures as an effective strategy for designing structural materials with exceptional performance over a wide temperature range [12, 13]. Interestingly, we found that in FCC high‐entropy alloys (HEAs), nanoscale coherent precipitation offers a unique opportunity to modulate recrystallization behavior. Discontinuous precipitates, which typically nucleate at grain boundaries, accelerate recrystallization by enhancing boundary mobility, whereas continuous precipitates, which form uniformly within grain interiors, exert strong Zener pinning and thus suppress recrystallization. Harnessing this contrast provides a powerful means to spatially control recrystallization activity, and consequently, microstructural topology. The primary focus of this study is to design a novel microstructural architecture through controlled precipitation modulation to achieve a strength–ductility synergy across a wide temperature range, while elucidating the underlying mechanisms responsible for the observed mechanical properties.

In this study, we introduce an innovative processing approach that combines cryo‐drawing with a dual‐aging strategy, coupling nanoscale precipitation with recrystallization to construct a bimodal harmonic architecture (BHA) in an FCC + L1_2_ HEA. Specifically, continuous precipitates stabilize coarse‐grained “cores”, while discontinuous precipitation‐assisted recrystallization generates necklace‐like fine‐grained “shells”, resulting in a topologically interconnected and highly reproducible microstructure. The resulting BHA alloy exhibits unprecedented mechanical properties across a wide temperature range, from −196 °C to 700 °C. The architecture not only achieves high strength and ductility at cryogenic and elevated temperatures but also effectively suppresses intermediate‐temperature embrittlement, a persistent challenge for precipitation‐strengthened HEAs and superalloys.

By integrating precipitation‐modulated recrystallization into microstructural design, this work establishes a new pathway for creating spatially programmed heterostructures with deterministic topology. The design principle provides a generalizable strategy for architecting hierarchical heterostructures in a wide range of alloys. Our findings demonstrate how bimodal nanoscale precipitation can be deliberately harnessed to guide recrystallization pathways, enabling the formation of a programmable, necklace‐like harmonic topology that is both structurally stable and functionally versatile. Our approach not only offers a novel solution to the strength–ductility trade‐off across a broad range of service temperatures, but also establishes an expandable paradigm for next‐generation structural materials required for extreme cryogenic and high‐temperature environments.

## 2 Results

### 2.1 Unique Harmonic Microstructure

**FIGURE 1:**
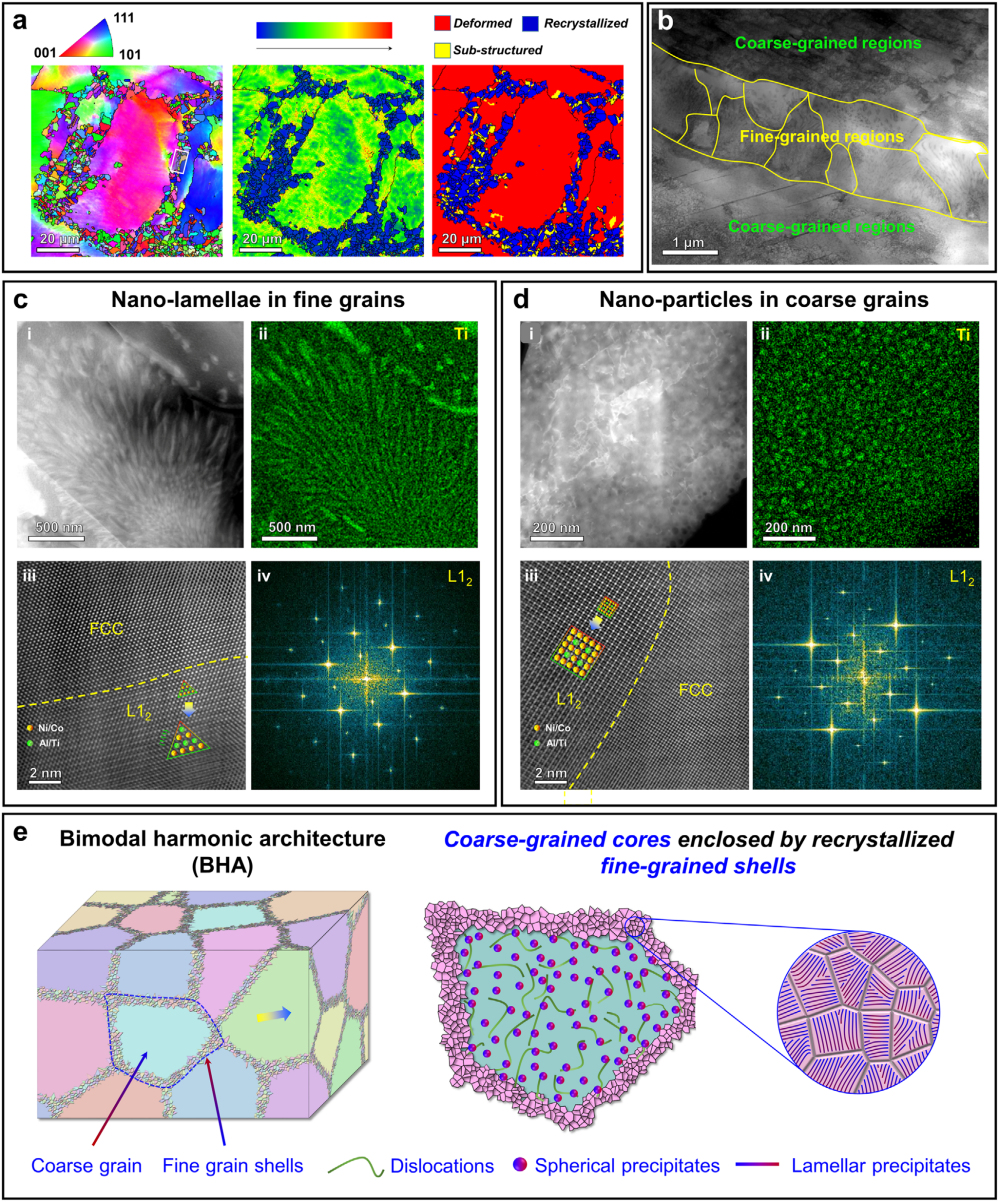
Necklace‐like grain architecture and dual‐mode precipitation in the BHA alloy. (a) EBSD IPF, KAM, and GOS maps of the microstructure. Coarse grains are enveloped by fine‐grained shells, forming a necklace‐like architecture. The fine‐grained shells exhibit reduced GND density and are fully recrystallized, whereas the coarse interiors remain non‐recrystallized. (b) TEM image highlighting chains of fine grains situated between coarse grains. (c) HR‐TEM and elemental mapping of fine grains, showing nanoscale lamellar discontinuous precipitates enriched in Ni, Al, and Ti. HR‐TEM and FFT patterns confirm their coherent L1_2_ structure. (d) TEM and EDS analysis of coarse grains, revealing spherical continuous precipitates distributed along dislocations. HR‐TEM and FFT confirm their L1_2_ lattice with sharp interfaces. (e) Schematic illustration of the BHA alloy, showing coarse grains encircled by fine grains. The fine grains contain lamellar discontinuous precipitates, while coarse grains host spherical continuous precipitates, highlighting the spatial coupling of grain topology and precipitate morphology.

We selected an alloy with a composition of (Ni_30_Co_25_Fe_25_Cr_20_)_91_Al_5_Ti_4_ (at.%) as a model system to demonstrate our microstructure engineering strategy. The alloy underwent a series of thermomechanical treatments, including solution treatment, cryogenic drawing, and subsequent two‐step aging. The microstructural evolution is summarized in Figure S1, which depicts the alloy in the solution‐treated, cryogenically drawn, single‐step aged, and two‐step aged states. The resulting BHA alloy exhibits a distinctive necklace‐like structure, as illustrated in Figure 1. Electron backscatter diffraction (EBSD) analysis highlights the hierarchical nature of this structure, as demonstrated by inverse pole figure (IPF), Kernel average misorientation (KAM), and grain orientation spread (GOS) maps (Figure 1a). The IPF map reveals a bimodal grain structure, with coarse grains (average size ∼100 µm) encircled by a continuous shell of fine grains (average size ∼1.5 µm), forming the distinctive necklace‐like morphology. The KAM map exhibits a pronounced contrast between the two regions: the fine‐grained shells exhibit significantly lower dislocation densities compared to the coarse‐grained interiors, suggesting substantial recovery within the shells during dual aging. Consistently, the GOS map confirms that the fine‐grained regions are fully recrystallized, whereas the coarse‐grained interiors remain in a non‐recrystallized state. Collectively, these observations indicate that selective recrystallization is localized near the original grain boundaries, where chemical and structural heterogeneities are most pronounced. Transmission electron microscopy (TEM) provides further confirmation of the necklace‐like harmonic structure (Figure 1b), revealing chains of fine grains (∼1.5 µm) aligned along the boundaries of coarse grains. Within these fine grains, lamellar precipitates are distinctly observed. High‐angle annular dark‐field scanning TEM (HAADF‐STEM) coupled with Ti mapping (Figure 1ci–ii) reveals that these lamellae are enriched in Ti, with an average width of approximately 30 nm. Energy‐dispersive spectroscopy (EDS) and atom probe tomography (APT) analyses (Figures S3a and S4a) further confirm enrichment in Ni, Al, and Ti, with a (Ni,Co):(Al,Ti) ratio of approximate 3:1. High‐resolution TEM (HR‐TEM) and fast Fourier transform (FFT) patterns (Figure 1ciii–iv) identify these precipitates as possessing an ordered L1_2_ lattice, consistent with discontinuous precipitates. The interfaces between the L1_2_ lamellae and the FCC matrix are largely indistinct, indicating strong coherency. In contrast, the interiors of coarse grains contain spherical continuous precipitates (∼24 nm in diameter) distributed along dislocations (Figure 1di, ii; Figures S3b and S4b). These precipitates exhibit a similar chemical composition to the lamellae, being enriched in Ni, Al, and Ti, while Fe, Co, and Cr remain in the matrix. They also adopt an L1_2_ structure and exhibit a nearly spherical morphology. (Figure 1diii–iv). The coexistence of coherent lamellar discontinuous precipitates in fine grains and spherical continuous precipitates in coarse grains highlights a dual‐mode precipitation pathway that is spatially coupled with the grain topology. Furthermore, we also examined the core–shell interfaces and observed no oxide particles or carbide phases (Figure S5).

This hierarchical configuration is schematically illustrated in Figure 1e. Coarse grains (∼100 µm) are encapsulated by shells of fine, recrystallized grains (∼1.5 µm), forming a 3D necklace‐like architecture. Within the coarse‐grained interiors, spherical continuous precipitates preferentially nucleate along deformation‐induced dislocations, whereas the fine shells accommodate lamellar discontinuous precipitates aligned along grain boundaries. This spatial partitioning of precipitation modes is governed by the distinct local microstructural states: near the original grain boundaries, recrystallization is coupled with discontinuous precipitation, whereas in the deformed grain interiors, the high dislocation density promotes continuous precipitation, which in turn suppresses recrystallization. This resulting synergy provides a robust microstructural foundation for the exceptional strength–ductility balance of the alloy over a wide temperature range.

### 2.2 Exceptional Mechanical Performance

**FIGURE 2:**
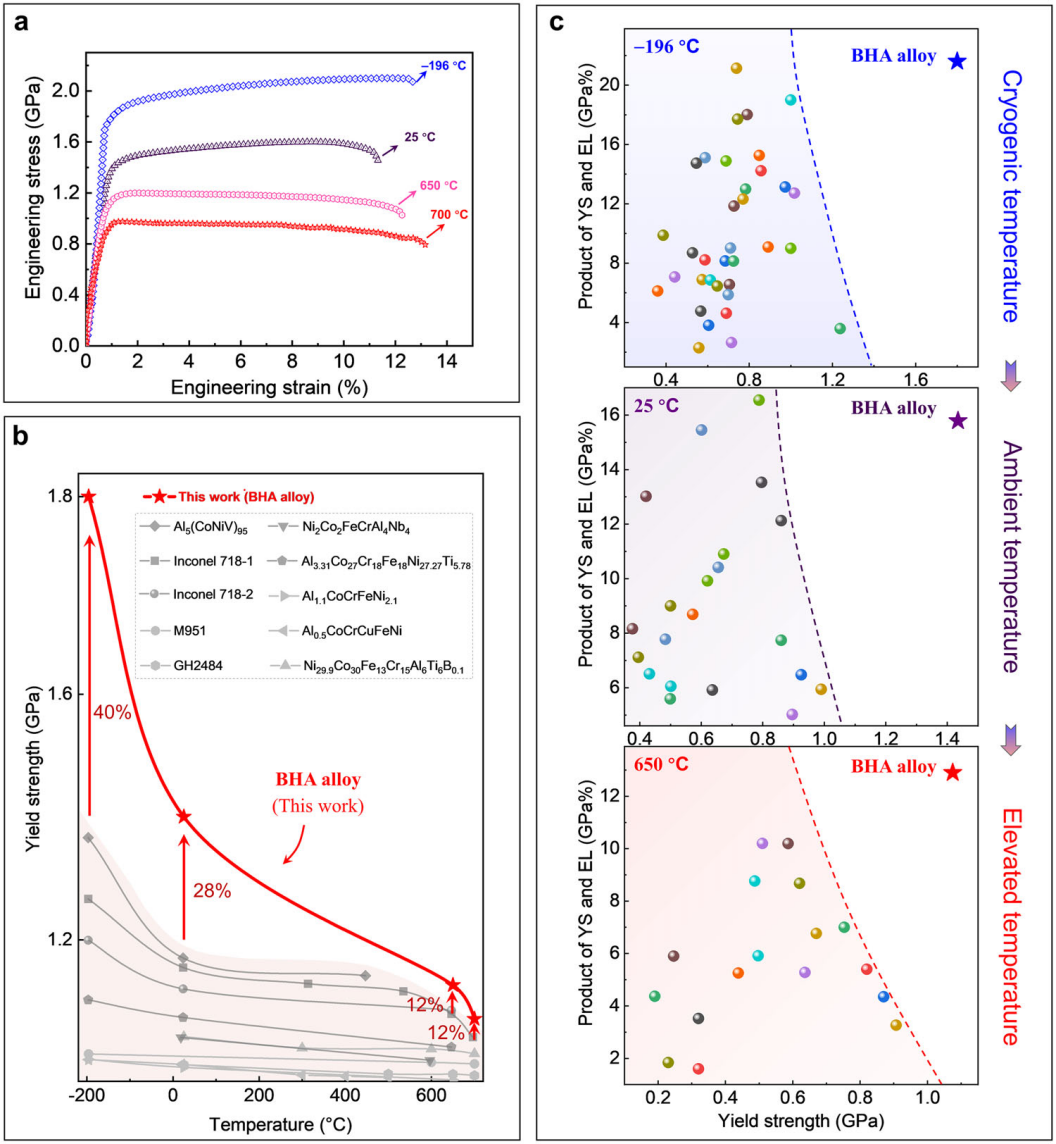
Superior mechanical properties of the BHA alloy across a wide temperature range. (a) Tensile stress–strain curves of the BHA alloy tested from −196 to 700 °C, showing an exceptional strength–ductility balance. (b) Temperature‐dependent yield strength compared with representative Ni‐based superalloys, steels, and FCC HEAs, highlighting the superior softening resistance of the BHA alloy. (c) Cross‐temperature yield strength × elongation (YS × EL) product vs. yield strength for the BHA alloy. In the cryogenic regime (−196 °C), the BHA alloy achieves YS × EL values exceeding 21 GPa·%. At room temperature (25 °C), the product remains above 16 GPa·%. Elevated at elevated temperatures (650 °C), the alloy maintains values above 12 GPa·%, significantly outperforming conventional structural alloys across all temperature regimes.

We systematically evaluated the tensile properties of the BHA alloy across a wide temperature range, from cryogenic conditions (−196 °C) to elevated temperatures up to 700 °C (Figure 2a). At −196 °C, the alloy exhibits a remarkable combination of ultrahigh strength and decent ductility, achieving a yield strength of 1800 ± 15 MPa, an ultimate tensile strength of 2100 ± 16 MPa, and a fracture elongation of 12% ± 1%. At room temperature, the BHA alloy continues to demonstrate excellent mechanical properties, with a yield strength of 1435 ± 12 MPa, an ultimate tensile strength of 1600 ± 15 MPa, and an elongation of approximately 11%, indicating that the cryogenic strengthening does not compromise ductility under ambient conditions. Remarkably, the alloy retains outstanding strength even at elevated temperatures, with yield strengths of 1075 ± 10 MPa at 650 °C and 950 ± 11 MPa at 700 °C, while sustaining elongations exceeding 12%; these values surpass those of most FCC‐based HEAs. These results clearly demonstrate the exceptional strength–ductility balance of our BHA alloy across a broad temperature range from −196 °C to 700 °C. To further clarify the specific role of the BHA, we compared the tensile properties of the BHA alloy at 650 °C with those of coarse‐grained (∼100 µm) and fine‐grained (∼10 µm) counterparts of identical composition and subjected to similar aging treatments (Figure S6). The results clearly demonstrate that the BHA alloy achieves a significantly improved strength–ductility synergy compared to both the coarse‐grained and fine‐grained samples under identical testing conditions, highlighting the distinct advantage of the harmonic structure in enhancing mechanical performance. To benchmark the BHA alloy against conventional materials, we compared its temperature‐dependent softening resistance with that of representative structural alloys [14, 15, 16, 17, 18, 19, 20, 21, 22, 23, 24] (Figure 2b). The results indicate that our BHA alloy consistently maintains superior yield strength across the entire temperature spectrum, significantly outperforming representative Ni‐based superalloys and high‐strength FCC alloys and demonstrating exceptional resistance to thermal softening. In particular, the BHA alloy exhibits overall superiority to the commercial Inconel 718 across the full temperature range, maintaining yield strengths approximately 40%, 28%, 12%, and 12% higher at −196, 25, 650, and 700 °C, respectively. To further assess its comprehensive performance, we compared the cross‐temperature mechanical performance of our BHA alloy with that of previously reported alloys (Figure 2c). Notably, at cryogenic (−196 °C), ambient (25 °C), and elevated (650 and 700 °C) temperatures, our BHA alloy achieves a consistently higher yield strength × elongation product compared to numerous reference alloys (see Supplementary Materials), while simultaneously retaining high yield strength. This ability to sustain yield strengths exceeding 900 MPa with ductility greater than 10% from cryogenic to elevated temperatures is exceptionally rare among structural alloys. The unique performance is attributed to the necklace‐like grain architecture and dual‐mode precipitation, which together stabilize strength while mitigating embrittlement. Collectively, these findings establish the BHA alloy as a transformative candidate for applications that demand simultaneous cryogenic, ambient, and high‐temperature performance—ranging from energy storage and transportation in cryogenic environments to next‐generation aerospace propulsion and other extreme‐environment structural components.

## 3 Discussion

### 3.1 Cooperative Strengthening Mechanisms across Temperatures

**FIGURE 3:**
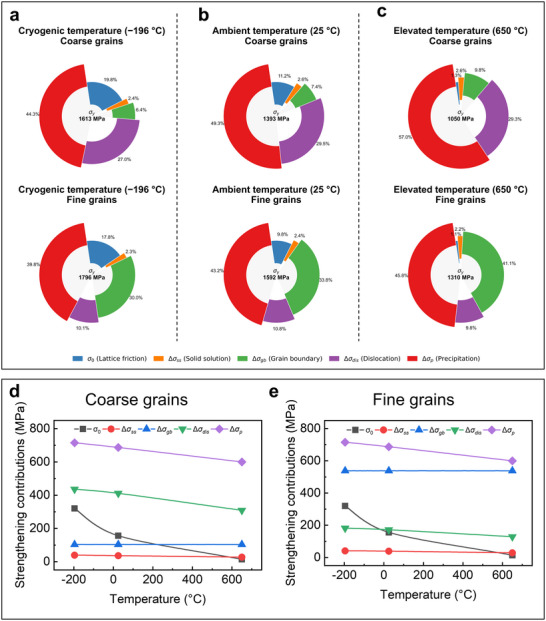
Quantitative analysis of strengthening mechanisms in the BHA alloy across different temperatures. (a–c) Relative contributions of lattice friction stress (*σ_0_*), solid‐solution strengthening (Δ*σ_ss_*), grain‐boundary strengthening (Δ*σ_gb_*), dislocation strengthening (Δ*σ_dis_*), and precipitation strengthening (Δ*σ_p_*) at cryogenic (**a**, −196 °C), ambient (**b**, 25 °C), and elevated (**c**, 650 °C) conditions for both coarse‐ and fine‐grained domains. (d), (e) Temperature‐dependent evolution of individual strengthening mechanisms for coarse grains (d) and fine grains (e). Precipitation and grain‐boundary strengthening remain the predominant contributors across all temperature regimes, while dislocation strengthening decreases sharply at elevated temperatures due to thermally activated recovery processes.

We first analyze the mechanisms underlying the exceptional strength of the BHA alloy across a wide range of temperatures. This remarkable performance fundamentally originates from the synergistic interplay between the necklace‐like structure and the dual‐mode nanoscale precipitates (spherical and lamellar). The yield point of the harmonic core–shell structured alloy lies between the yield strength of the coarse‐grained core and that of the fine‐grained shell, being elevated above the core's yield strength due to hetero‐deformation‐induced (HDI) strengthening but still below the shell's yield strength. These microstructural features contribute in a temperature‐dependent yet complementary manner, enabling the alloy to maintain an outstanding strength–ductility synergy from cryogenic to elevated temperatures. To unravel the origin of strength across the wide temperature range, we quantitatively evaluated the relative contributions of each mechanism using established strengthening models. The strengthening behavior of the BHA alloy was systematically quantified at cryogenic, ambient, and elevated temperatures, with the results summarized in Figure 3. Both the strengthening contribution diagrams (Figure 3a–c) and the quantitative plots (Figure 3d, e) reveal the distinct, temperature‐dependent evolution of individual strengthening contributions, including lattice friction stress (*σ*_0_), solid‐solution strengthening (Δ*σ_ss_*), grain‐boundary strengthening (Δ*σ_gb_*), dislocation strengthening (Δ*σ_dis_*), and precipitation strengthening (Δ*σ_p_*). At cryogenic temperature (−196 °C), precipitation strengthening emerges as the predominant mechanism in both coarse‐ and fine‐grained domains, accounting for approximately 44% and 40% of the total strengthening, respectively (Figure 3a). In coarse grains, dislocation forest hardening is the second most significant contributor, providing a remarkable increment of 436 MPa (27% of the total strength). In contrast, in fine grains, the Hall–Petch strengthening (Δ*σ_gb_* ≈ 538 MPa, ∼30% of the total strength) serves as the secondary mechanism. The combined effects of coherent precipitate shearing and dynamic dislocation barriers result in the ultrahigh yield strength of ∼1800 MPa observed under cryogenic conditions. At ambient temperature (25 °C), the relative contributions of the various strengthening mechanisms become more balanced (Figure 3b). In coarse grains, precipitation strengthening (49%) and dislocation strengthening (29%) remain the primary contributors, while grain‐boundary and solid‐solution strengthening together account for less than 12% of the total strength. In fine grains, precipitation strengthening (43%) and grain‐boundary strengthening (34%) play significant roles, while dislocation strengthening decreases markedly (∼11%). This distribution highlights the synergistic effect of coherent nanoscale precipitates and the necklace‐like shells: the precipitates serve as strong obstacles to dislocation motion in coarse grains, while the fine‐grained shells offer stable Hall–Petch barriers. Consequently, the alloy maintains a yield strength of approximately 1400–1600 MPa at room temperature. At elevated temperature (650 °C), precipitation strengthening remains the predominant mechanism, contributing approximately 57% in coarse grains and 46% in fine grains (Figure 3c). In contrast, dislocation strengthening drops sharply to approximately 29% in coarse grains and 10% in fine grains, primarily due to thermally activated recovery processes. Notably, grain‐boundary strengthening remains essentially unchanged with temperature (∼103 MPa in coarse grains and ∼538 MPa in fine grains), thereby ensuring structural stability of the necklace‐like shells. The quantitative trends in Figure 3d, e further demonstrate that the lattice friction stress (*σ_0_*) decreases markedly with increasing temperature, while both precipitation strengthening (Δ*σ_p_*) and dislocation strengthening (Δ*σ_dis_*) gradually diminish. In contrast, grain boundary strengthening (Δ*σ_gb_*) and solid‐solution strengthening (Δ*σ_ss_*) remain relatively stable, effectively mitigating the severe softening typically observed in conventional FCC HEAs and Ni‐based superalloys. This balanced interplay of mechanisms accounts for the BHA alloy's ability to retain yield strengths above 1 GPa with ductility exceeding 12% even at 650 °C. In addition, loading–unloading–reloading (LUR) tests were performed at −196 °C, 25 °C, and 650 °C to quantify the extra HDI stress associated with the BHA, as shown in Figure S7. The measured HDI stresses under these conditions are 905, 752, and 626 MPa, respectively. These results are consistent with our strengthening analysis, which demonstrates that the mechanical contrast between the core and shell regions enhances hetero‐deformation incompatibility, thereby intensifying the HDI effect. It should be noted that the physical interpretation of the additional stress measured in LUR tests remains a subject of ongoing debate [25, 26, 27]; therefore, we did not quantitatively separate HDI hardening from other strengthening mechanisms in this study.

Overall, the strengthening mechanisms of the BHA alloy demonstrate a distinct temperature‐adaptive synergy. At cryogenic temperatures, the strength of the alloy is primarily governed by dislocation forest hardening and precipitate shearing. At ambient temperature, both precipitation and grain‐boundary strengthening make substantial contributions, thereby supporting sustained high yield strength. At elevated temperatures, the presence of stable nanoscale precipitates and persistent grain‐boundary strengthening becomes increasingly dominant, effectively compensating for the reduction in dislocation hardening. This cooperative evolution of strengthening mechanisms underpins the exceptional strength of the BHA alloy across a wide temperature range, establishing a robust structural foundation for its broad‐temperature applicability.

### 3.2 Adaptive Deformation Mechanisms across Temperatures

**FIGURE 4:**
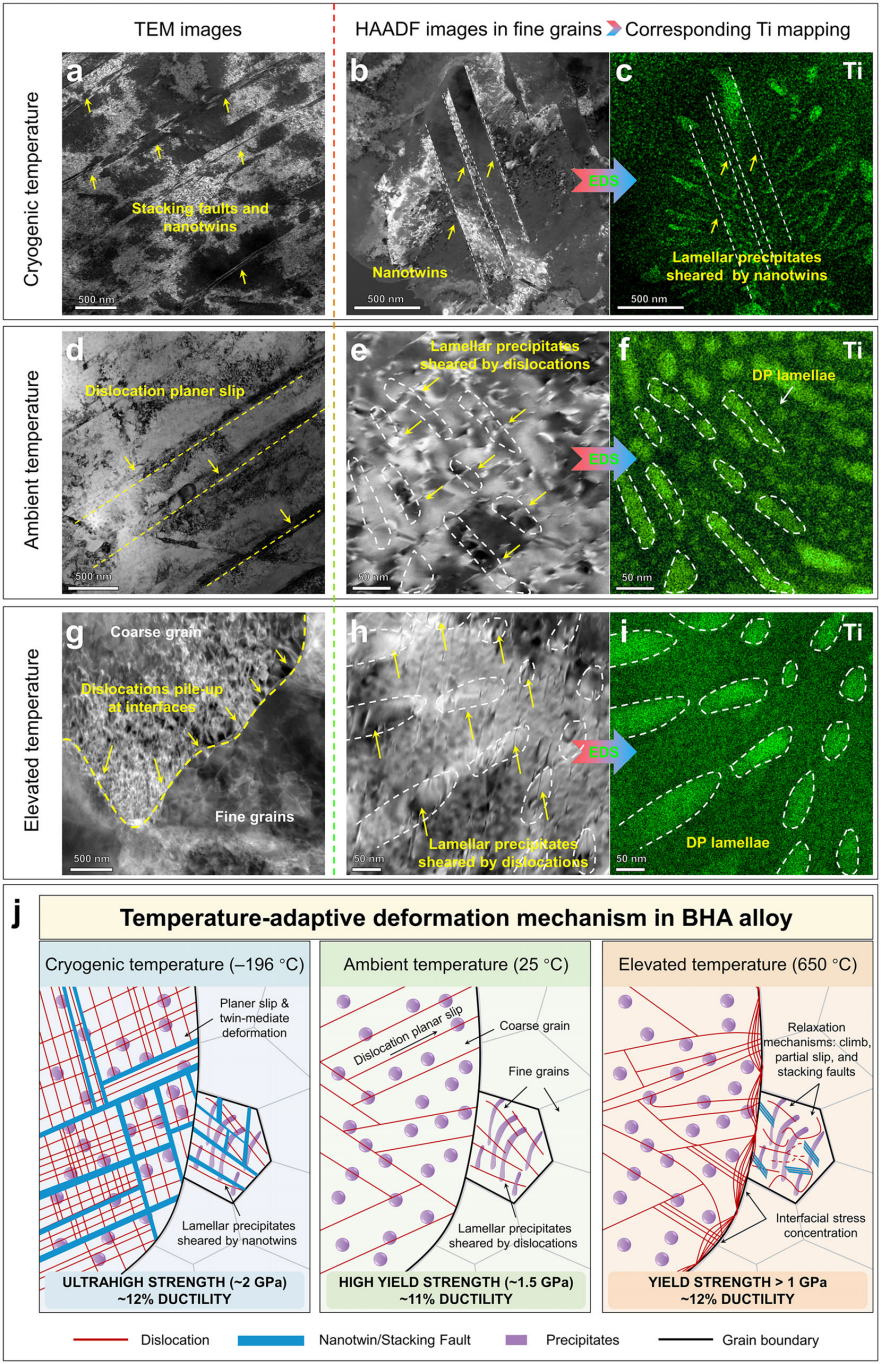
Deformation mechanisms of the BHA alloy at different temperatures. (a–c) At cryogenic temperature (−196 °C): the formation of dislocation forests, nanotwins formation, and twin–precipitate interactions collectively enhance strain hardening. (d–f) At ambient temperature (25 °C): deformation is predominated by dislocation slip, with extensive shearing of lamellar precipitates by dislocations. (g–i) At elevated temperature (650 °C): dislocation pile‐ups at core–shell interfaces generate stress concentration that activates relaxation mechanisms, thereby facilitating coordinated deformation between coarse and fine grains and preserving ductility. (j). Schematic diagram of temperature‐adaptive deformation mechanisms in the BHA alloy.

The BHA alloy exhibits remarkable adaptability across cryogenic, ambient, and elevated temperatures, which enables sustained ductility under diverse conditions. To elucidate the mechanisms underlying this impressive ductility across a wide temperature range, we examined the deformation microstructures at various temperatures using TEM. At cryogenic temperature (−196 °C), plastic deformation is primarily governed by planar slip coupled with dislocation–precipitate interactions, while twin‐mediated mechanisms contribute additional plastic accommodation. In coarse grains (Figure 4a), dense dislocation networks, stacking faults, and nanotwins are observed, forming strong barriers that enhance work hardening. Simultaneously, nanotwins are frequently activated (Figure 4b) in fine grains, providing additional obstacles to dislocation motion and facilitating plastic deformation. Figure 4c and Figure S8 further show that the lamellar precipitates are sheared by nanotwins, indicating their compatibility with twin‐mediated deformation. Although twinning is generally less favorable in fine grains due to the increase in critical twinning stress with decreasing grain size [28, 29], the harmonic core–shell architecture creates a distinctly different local stress state. The softer core deforms more readily, whereas the harder shell sustains a strong interfacial constraint caused by strain incompatibility. This leads to pronounced local stress concentrations within the shell, enabling deformation twinning to be activated once the local stress exceeds the critical twinning stress [30]. To gain deeper insight into the origin of twinning, we estimated the stacking fault energy (SFE) using the thermodynamic model proposed by Olson et al. [31]. (see Supplementary Information for details) The results show that the matrix SFE decreases significantly after precipitation, dropping from ∼109 mJ/m^2^ in the solution‐treated state to about 12–13 mJ/m^2^ at −196 °C, which strongly facilitates the activation of deformation twinning under cryogenic conditions. This cooperative interplay among dislocations, nanotwins, and precipitate shearing enhances strain hardening and prevents premature strain localization, allowing the alloy to achieve ultrahigh strength together with approximately 12% elongation at cryogenic conditions. At ambient temperature (25 °C), deformation is primarily governed by dislocation planar slip, with significant dislocation–precipitate interactions. In coarse grains (Figure 4d), clear evidence of dislocation glide is observed, while in the fine‐grained shells (Figure 4e), lamellar precipitates are extensively sheared by dislocations. Ti elemental mapping (Figure 4f) confirms that the precipitates retain their lamellar morphology and coherency during deformation, ensuring stable precipitation strengthening while facilitating effective slip transfer across interfaces. The simultaneous operation of precipitate shearing and grain‐scale strain partitioning provides steady strain‐hardening capability, enabling the alloy to sustain a high yield strength of approximately 1400–1600 MPa while preserving approximately 11% ductility at room temperature. At elevated temperature (650 °C), the deformation behavior diverges from that of conventional alloys that typically suffer from intergranular embrittlement. Coarse‐ and fine‐grained domains are clearly distinguished (Figure 4g), with pronounced dislocation pile‐ups at the core–shell interfaces. These pile‐ups generate significant stress concentration; however, rather than leading to intergranular cracks, they activate auxiliary relaxation mechanisms within the fine shells, including dislocation climb, partial slip, and localized stacking faults (Figure S9). Furthermore, we did not observe significant grain boundary sliding in the fine‐grained shell (Figure S10), likely due to the deformation temperature being insufficient to activate grain boundary mechanisms. Meanwhile, lamellar precipitates remain coherent and continue to be sheared by dislocations (Figure 4i), underscoring their exceptional stability. The interfacial stress concentration promotes coordinated deformation between coarse and fine grains, effectively redistributing strain and suppressing crack initiation. This mechanism of interfacial stress accommodation explains why the alloy retains ductility above 12% while maintaining yield strengths exceeding 1 GPa at 650 °C.

Consequently, our BHA alloy demonstrates a distinct temperature‐adaptive synergy in its plasticity mechanisms (Figure 4j). At cryogenic temperature, dislocation slip and precipitate shearing remain dominant, while forest dislocations and nanotwins further enhance work hardening. At room temperature, plasticity is sustained through dislocation slip and precipitate shearing, leading to a well‐balanced combination of strength and ductility. At elevated temperature, interfacial stress concentration activates additional relaxation pathways, enabling strain redistribution and preventing intergranular fracture. Overall, the synergistic interplay of dislocation–precipitate interactions, HDI hardening, and temperature‐adaptive deformation mechanisms endows the BHA alloy with exceptional strength–ductility synergy across a broad temperature range, conferring outstanding resistance to embrittlement and ensuring reliable performance under diverse service conditions.

## 4 Conclusions

We present an innovative alloy design strategy based on the engineering of necklace‐like bimodal harmonic architecture, which imparts exceptional mechanical robustness to the alloy across a broad temperature range (−196 to 700 °C), consistently achieving yield strengths of 1–2 GPa and ductilities exceeding 10%. Our findings reveal that precipitation and grain‐boundary strengthening are the predominant strengthening mechanisms across all temperature regimes, while the deformation mechanisms exhibit a dynamic and synergistic adaptation, encompassing dislocation slip, nano‐twinning, dislocation–precipitate interactions, and hetero‐deformation. The temperature‐adaptive synergy between strengthening and deformation mechanisms effectively mitigates cryogenic embrittlement and elevated‐temperature softening, thereby ensuring reliable mechanical performance under extreme conditions. The concept of bimodal harmonic architecting represents a transformative and broadly applicable paradigm for the development of next‐generation alloys with superior mechanical properties across diverse temperature environments.

## 5 Experimental Section

### 5.1 Alloy Preparation

An ingot with a nominal composition of (Ni_30_Co_25_Fe_25_Cr_20_)_91_Al_5_Ti_4_ (at.%) was prepared using a 25‐kg vacuum induction melting furnace under a high‐purity argon atmosphere. Raw metals (purity greater than 99.5 wt.%) were employed to fabricate the target alloy. The as‐cast ingot with dimensions of ϕ80 × 200 mm^3^ was homogenized at 1150 °C for 2 h, followed by water quenching. The homogenized ingot was hot forged at 1100 °C into a rod with a diameter of 25 mm. After annealing at 1100 °C, bars with a diameter of 10 mm were machined from the forged rod for subsequent cryogenic drawing. Multi‐pass cryogenic drawing was carried out to obtain a bar with an equivalent strain of 0.71 (reducing the diameter from 10 to 7 mm, estimated by the equation *ε =* 2*ln*(*d_0_/d*) [32]). Prior to each pass, samples were immersed in a liquid nitrogen bath for at least 10 min to maintain the deformation temperature at ‒196 °C. The BHA alloy was prepared through a two‐step aging after cryogenic drawing: the cryogenically drawn bar was isothermally heated at 900 °C for 10 min, then immediately transferred to a furnace at 750 °C and aged for 4 h, followed by water quenching (referred to as the BHA alloy).

### 5.2 Mechanical Testing

Dog‐bone‐shaped specimens with a gauge of 15 mm and a diameter of 3 mm were machined from the BHA alloy for tensile testing at cryogenic, ambient, and elevated temperatures. Uniaxial tensile tests were carried out at various temperatures using a Z100 universal testing machine at a strain rate of 10^−3^ s^−1^. Strain measurements were obtained using a laser extensometer to ensure high precision under all temperature conditions. For each testing condition, three samples were tested to ensure the accuracy and reliability of the results.

### 5.3 Microstructural Characterization

EBSD measurements were performed using an FEI Apreo field‐emission environmental SEM equipped with an EBSD detector and an automatic orientation acquisition system (Oxford Instruments‐HKL Channel 5). Samples for SEM and EBSD were mechanically polished and subsequently electropolished in a 10% oxalic acid aqueous solution at 20 V and 0.05 A for 10 min at room temperature. TEM and STEM characterizations were performed on an FEI Talos F200i microscope operated at 200 kV. Discs with a diameter of 3 mm for TEM and STEM were cut from the longitudinal section near the tensile fracture and then were mechanically polished to a thickness of 40 µm, followed by twin‐jet polishing to a thickness of electron transparency in a solution consisting of 10 vol.% perchloric acid and 90 vol.% ethanol at ‒25 °C. APT measurements were conducted using a local electrode atom probe (CAMECA LEAP 5000XR) in voltage‐pulsed mode, operated at ‒223 °C with a pulse rate of 200 kHz and a pulse fraction of 20%. Needle‐shaped specimens for APT measurements were prepared by lift‐outs and annular milled in an FEI Scios focused ion beam/SEM (FIB/SEM) system.

## Conflicts of Interest

The authors declare no conflicts of interest.

## Supporting information




**Supporting File**: advs75356‐sup‐0001‐SuppMat.docx.

## Data Availability

The data that support the findings of this study are available from the corresponding author upon reasonable request.
