# Supplementary material for: Precipitation‐Modulated Harmonic Architectures Enable Superior Strength–Ductility Synergy from Cryogenic to Elevated Temperatures in Nanostructured Alloys
Source: Adv Sci (Weinh). 2026 Apr 23;13(39):e75356. doi: 10.1002/advs.75356 (PMC13335530; doi:10.1002/advs.75356)
Supplement: Supplementary file 1 — Supporting File: advs75356‐sup‐0001‐SuppMat.docx. [file ADVS-13-e75356-s001.docx]

**Supplementary materials for**

**Precipitation-modulated harmonic architectures enable superior strength–ductility synergy from cryogenic to elevated temperatures in nanostructured alloys**

W. Li^a,b^, L.Y. Xiong^a,c,*^, M.C. Niu^a^, J.H. Luan^d^, W. Wang^a,c,*^, Z.B. Jiao^b,e,*^

*^a^ Institute of Metal Research, Chinese Academy* *of Sciences, Shenyang 110016, China;*

*^b^ Department of Mechanical Engineering, Research Institute for Advanced Manufacturing, The Hong Kong Polytechnic University, Hong Kong, China;*

*^c^ School of Materials Science and Engineering, University of Science and Technology of China, Shenyang 110016, China;*

*^d^ Inter-university 3D Atom Probe Tomography Unit, Center for Advanced Nuclear Safety and Sustainable Development, City University of Hong Kong, Hong Kong, China*

*^e^ The Hong Kong Polytechnic University Shenzhen Research Institute, Shenzhen 518057, China;*

*^*^ Corresponding authors: [lyxiong@imr.ac.cn](mailto:lyxiong@imr.ac.cn) (L.Y.X);* [*wangw@imr.ac.cn*](mailto:wangw@imr.ac.cn) *(W.W.);* [*zb.jiao@polyu.edu.hk*](mailto:zb.jiao@polyu.edu.hk) *(Z.B.J.)*

## Note 1. Microstructural evolution

In the solution-treated condition, the alloy exhibits a single FCC phase (**Fig. S1a**) with fully recrystallized equiaxed grains averaging 96 ± 6 μm in size, as illustrated in the inverse pole figure (IPF) map. Severe cryogenic drawing at −196 °C elongates these equiaxed grains and introduces a high density of dislocations and slip bands within their interiors (**Fig. S1b**). A brief annealing at 900 °C for 10 min (**Fig. S1c**) promotes the localized recrystallization along the boundaries of the deformed grains, leading to the formation of numerous fine grains in these regions. Meanwhile, as further evidenced by the STEM–EDS mapping results in **Fig. S2**, lamellar discontinuous precipitates are formed within the recrystallized grains at this stage, suggesting a coupled interaction between discontinuous precipitation and recrystallization. Subsequent aging at 750 °C preserves this microstructure topology while further stabilizing the necklace-like architecture, ultimately resulting in the BHA alloy.

## Note 2. Strengthening mechanism

*Strengthening mechanism at room temperature*

We attribute the excellent strength of our BHA alloy to the synergistic effects of nanoscale precipitates, dislocations, and hetero-structures. To quantitatively evaluate the contribution of these structures, we calculated the theoretical strength of both the fine- and coarse-grained domains at room temperature. The overall yield strength of these domains was estimated by the arithmetic addition (Eq. S1) of individual strengthening contributions, including grain boundary strengthening (Δ*σ_gb_*), solid-solution strengthening (Δ*σ_ss_*), dislocation strengthening, (Δ*σ_dis_*), precipitation strengthening (Δ*σ_p_*)

| $\sigma_{y}=\sigma_{0}+\Delta\sigma_{ss}+\Delta\sigma_{gb}+\Delta\sigma_{dis}+\Delta\sigma_{p}$ | (S1) |
| --- | --- |

where *σ_0_* is the lattice fraction strength of the CoCrFeNi matrix, which was estimated to be ~156 MPa ^1^.

For the calculation of solid solution strengthening (Δ*σ_ss_*), Al and Ti were considered as solute elements in the CoCrFeNi alloy. The strengthening contribution was estimated using the Fleischer model ^2^

| $\Delta\sigma_{ss}=M\frac{G\varepsilon_{s}^{3/2}c^{1/2}}{700}$ | (S2) |
| --- | --- |

where *c* is the concentration of solute atoms, and *ε_s_* the effective misfit parameter given by

| $\varepsilon_{s}=\left\vert\frac{\varepsilon_{G}}{1+0.5\varepsilon_{G}}-3\varepsilon_{a}\right\vert$ | (S3) |
| --- | --- |

where *ε_a_* is the size misfit parameter, and *ε_G_* is the modulus misfit parameter. According to Gwalani *et al.* ^3^, the modulus misfit term can be negligible compared to the size misfit in high-entropy alloys (HEAs), allowing the approximation

| $\varepsilon_{s}=\left\vert3\varepsilon_{a}\right\vert=\left\vert\frac{3}{a_{0}}\frac{\Delta a}{\Delta c}\right\vert$ | (S4) |
| --- | --- |

where *a_0_* = 0.360 nm is the lattice parameter of the CoCrFeNi base alloy ^4^, and *a_alloy_* = 0.363 nm is the lattice parameter of the current alloy, as determined by SAED analysis. Using the measured concentration of Al and Ti from Table S1, the solid solution strengthening contributions in the fine- and coarse-grained domains were estimated to be approximately 39 and 36 MPa, respectively.

The contributions of Δ*σ_gb_* and Δ*σ_dis_* were estimated through the following equations ^5^

| $\Delta\sigma_{gb}=kd^{-1/2}$ | (S5) |
| --- | --- |
| $\Delta\sigma_{dis}=M\alpha Gb\sqrt{\rho}$ | (S6) |

where *k* = 840 MPa∙μm^1/2^ represents the strengthening coefficient ^6, 7^, and *d* is the average grain size, which can be evaluated from on the EBSD analysis of the two domains. To distinguish the fine- and coarse-grained domains, the grain orientation spread (GOS) was used to screen recrystallized and non-recrystallized grains. Grains with GOS < 2.5° were identified as recrystallized grains, while those with GOS ≥ 2.5° were classified as non-recrystallized grains ^8^. The screened subsets are presented in **Fig. S11**. Based on these screened subsets, the average grain size of the fine-grained regions was determined to be 2.4 µm. Gvien that the coarse-grained regions are non-equiaxed, the directional intercept method was employed to determine an “effective grain size” (*d_eff_*) of 66 µm in these regions ^9^. The Taylor factor (*M*) for FCC alloys is 3.06. The shear modulus (*G*) of the CoCrFeNi HEA is 84 GPa^10^, and the Burgers vector (𝑏) of the current alloy, obtained through SAED patterns, is 0.257 nm. The constant *α* = 0.3 is an average value for polycrystalline FCC materials ^11^. Due to the challenges inherent in directly measuring the total dislocation densities within different domains, the geometrically necessary dislocation (GND) density was used as a proxy. According to the strain gradient model ^12, 13^, the GND density can be estimated by

| $\rho_{GND}=\frac{2\Delta\theta}{\mu b}$ | (S7) |
| --- | --- |
| $\Delta\theta=\frac{\sum_{j=1}^{n} \left\vert\theta_{j}^{sur}-\theta_{i}\right\vert}{n}$ | (S8) |

where *θ_i_* is the misorientation angle of point *i*, *θsur j* is the local misorientation angle of adjacent point *j*, *ρ_GND_* is the GND density, Δ*θ_i_* represents the local misorientation angle, *μ* is the unit length, and *b* is the magnitude of Burger's vector (0.257 nm for the current alloy). Using the GOS subset (with a critical value of 2.5°), the GND densities for the fine- and coarse-grained domains were estimated to be 7.49×10^13^ and 4.29×10^14^ m^–2^, respectively. Based on these results, the grain boundary strengthening increments (Δ*σ_gb_*) were calculated to be 538 MPa for the fine-grained region and 245 MPa for the coarse-grained regions. The dislocation strengthening increments (Δ*σ_dis_*) were calculated to be 171 MPa for the fine-grained regions and 410 MPa for the coarse-grained regions.

To evaluate the contribution of precipitation strengthening, the precipitate shearing mechanism was used to calculate the contribution of continuous precipitates in the coarse-grained domains and discontinuous precipitates in the fine-grained domains ^3, 14^

| $\Delta\sigma_{CPs}=0.81M\frac{\gamma_{APB}}{2b}(\frac{3\pi f}{8})^{1/2}$ | (S9) |
| --- | --- |

where *γ_APB_=*0.268 J/m^2^ is the antiphase boundary (APB) energy taken from Ni-based superalloys ^15^, and *f* = 24% is the total volume fraction of both continuous and discontinuous precipitates, determined using the lever rule method. Accordingly, the strengthening increment contributed by continuous and discontinuous precipitates was calculated to be 687 MPa.

Based on our modeling, the strengthening mechanisms in the bimodal domains can be attributed to the three primary features: dislocations, grain boundaries, and nano-precipitates (**Fig. 3**). The coarse-grained domains retain a significant number of dislocations induced by cryogenic drawing, leading to a higher strength increment of 411 MPa compared to 172 MPa in the fine-grained domains. Regarding grain boundary strengthening, the fine-grained domains exhibit a substantial strengthening effect of 538 MPa, surpassing the 103 MPa observed in the coarse-grained domain. Furthermore, due to the high APB energy, both continuous and discontinuous precipitates contribute significantly to the overall strengthening, with comparable increments of approximately 687 MPa. It can be seen that the estimated room temperature yield strength of the fine-grained domains (1592 MPa) is significantly higher than that in the coarse-grained domains. By applying the principle of linear superposition (using volume fractions of 5% for the fine-grained domains and 95% for coarse-grained domains, as determined from EBSD results), the theoretical yield strength of the BHS alloy was calculated to be approximately 1403 MPa, which is in good agreement with our experimental results (1435 ± 12 MPa). Thus, the extraordinary room-temperature strength of the BHS alloy can be attributed to the synergistic effects of multiple strengthening mechanisms, including the presence of bimodal precipitates, a high density of dislocations, and recrystallized fine grains at the original grain boundaries.

*Strengthening mechanism at cryogenic and high temperatures*

At cryogenic and elevated temperatures, the temperature-dependence of each strengthening mechanism should be considered. Accordingly, Eq. S1 is modified to incorporate the temperature-dependent parameters

| $\sigma_{y}(T)=\sigma_{0}(T)+\Delta\sigma_{ss}(T)+\Delta\sigma_{gb}(T)+\Delta\sigma_{dis}(T)+\Delta\sigma_{p}(T)$ | (S10) |
| --- | --- |

The intrinsic lattice friction stress, *σ*_0_(*T*), can be estimated using a temperature-dependent relation ^7^

| $\sigma_{0}(T)=\sigma_{0}(T_{0})\exp(\frac{-2\pi\omega_{0}}{bT_{m}}T)$ | (S11) |
| --- | --- |

where *σ*_0_(*T*_0_) = 426 MPa is the lattice friction stress at 0 K (–273 ℃), *T* is the testing temperature, *T_m_* is the melting point of the alloy, and *ω*_0_ is the dislocation width at 0 K, and *ω*_0_ = *b* (Burgers vector) is adopted according to the study of Wu *et al.* ^7^. Based on this model, *σ*_0_(*T*) was estimated to be approximately 320 MPa at –196 °C and 14 MPa at 650 °C.

Previous studies on FCC HEAs, such as CoCrFeMnNi and CoCrFeNi ^7, 16^, show that the Hall–Petch slope remains largely temperature-independent. Thus, grain boundary strengthening (*σ_gb_*) is assumed constant across the examined temperature range. Solid solution strengthening (Δ*σ_ss_*(*T*)) and dislocation strengthening (Δ*σ_dis_*(*T*)) depend on the shear modulus (*G*(*T*)). Naeem *et al*. ^17^ provided an empirical model for *G*(*T*) in the CoCrFeNi alloy

| $G(T)=G_{0}-\frac{A}{[\exp(\frac{B}{T})-1]}$ | (S12) |
| --- | --- |

The vales of fitting parameters, *G*_0_ =89.4 GPa, *A* = 13, and *B* = 373, are taken from the study of Laplanche *et al*. ^18^. This yields shear modulus values of approximately 89 GPa at –196 °C and 63 GPa at 650 °C, which were used in Eqs. S2 and S6 to calculate Δ*σ_ss_*(*T*) and Δ*σ_dis_*(*T*), respectively.

At –196 °C and 650 °C, precipitation strengthening (Δ*σ_p_*(*T*)) was modeled using Eq. S9, incorporating the temperature effects through the APB energy *γ*_APB_(*T*), as estimated by ^19^

| $\gamma_{APB}(T)=\gamma_{APB}(T_{0})\frac{f(T)}{f(T_{0})}(\frac{1+\int_{o}^{T_{0}} \alpha(T)dT}{1+\int_{o}^{T} \alpha(T)dT})^{2}(1-\frac{\int_{T_{0}}^{T} C_{v}(T)dT}{\int_{T_{0}}^{T_{B}} C_{v}(T)dT+\Delta H_{M}+\Delta H_{B}})$ | (S13) |
| --- | --- |

where *γ_APB_*(*T*_0_) = 0.268 J/m^2^ is the APB energy at room temperature, and *f*(*T*) is the volume fraction of the L1_2_ phase, which was assumed to remain constant (*f*(*T*) = *f*(*T*_0_)). The linear expansion coefficient *α*(*T*) and isochoric specific heat *C_v_*(*T*) are obtained from JmatPro

| $\alpha(T)=4.20\times10^{-9}T+14.20\times10^{-6}(1/K)$ | (S14) |
| --- | --- |
| $C_{v}(T)=9.36\times10^{-3}T+24.32(J/mol\cdot K)$ | (S15) |

The calculated *γ*_APB_(*T*) values (Table S5) were substituted into Eq. S9 to calculate Δ*σ_p_*(*T*) at –196 °C and 650 °C (see Table S6).

The total strengthening increments at various temperatures are summarized in Table S6. The results show that the yield strength of the fine-grained regions exceeds that of the coarse-grained regions by 183 MPa at –196 °C and 265 MPa at 650 °C. Using linear superposition, the theoretical yield strengths of the BHA alloy were estimated to be 1622 MPa at –196 °C and 1063 MPa at 650 °C.

## Note 3. Estimation of stacking fault energy

The stacking fault energy was estimated using the Olson–Cohen thermodynamic model ^20, 21^, which treats a stacking fault as a thin HCP embryo bounded by two FCC/HCP interfaces. The ideal SFE is given by

| $\gamma_{\text{SFE}}=2\rho\Delta G^{\gamma\to\varepsilon}+2\sigma$ | (S16) |
| --- | --- |

Where *ρ* is the molar surface density of the {111} close-packed plane, Δ*G^γ→ε^* is the molar Gibbs free-energy difference for the FCC to HCP transformation, and *σ* is the interfacial energy between the HCP embryo and the FCC matrix. The molar surface density was calculated as

| $\rho=4/\sqrt{3}a^{2}N_{\text{A}}$ | (S17) |
| --- | --- |

where *a* is the FCC lattice parameter and *N_A_* is Avogadro’s constant. The Gibbs free-energy difference was evaluated by

| $\Delta G^{\gamma\to\varepsilon}=G_{m}^{\text{HCP}}-G_{m}^{\text{FCC}}$ | (S18) |
| --- | --- |

where *G*_m_^HCP^ and *G*_m_^FCC^ are the molar Gibbs free energies of the HCP and FCC phases, respectively, at the same composition and temperature. In this study, these values were obtained using Thermo-Calc with the TCHEA4 database. For the solution-treated state, the nominal alloy composition was used. For the post-precipitation condition, the matrix compositions in the coarse-grained and fine-grained regions after precipitation were used for the calculation. Here, an interfacial energy of 8 mJ/m^2^ was used according to Curtze *et al*. ^22^. The calculated results show that the SFE of the present alloy in the solution-treated state is approximately 109 mJ/m^2^ at −196 °C, whereas after precipitation, the SFE of the matrix decreases markedly to about 12 mJ/m^2^ and 13 mJ/m^2^ for the coarse-grained and fine-grained regions, respectively. This substantial reduction indicates that precipitation-induced solute partitioning significantly lowers the matrix SFE, thereby strongly facilitating the activation of deformation twinning under cryogenic conditions.


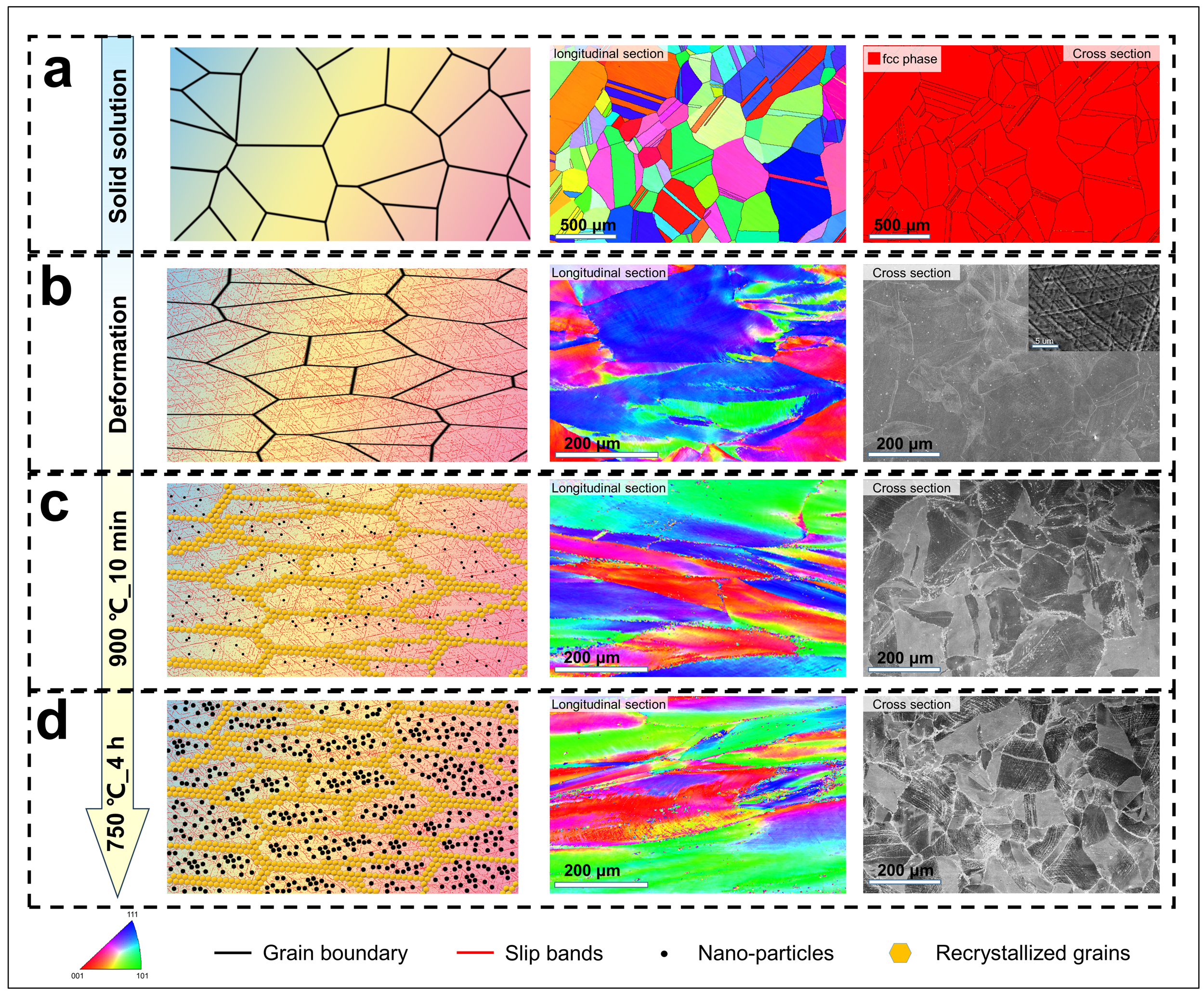


**Figure. S1**. Microstructure evolution of the alloy at various stages: **a.** solution-treated, **b.** cryogenic-drawn, **c.** first-step aged, and **d.** two-step aged conditions.


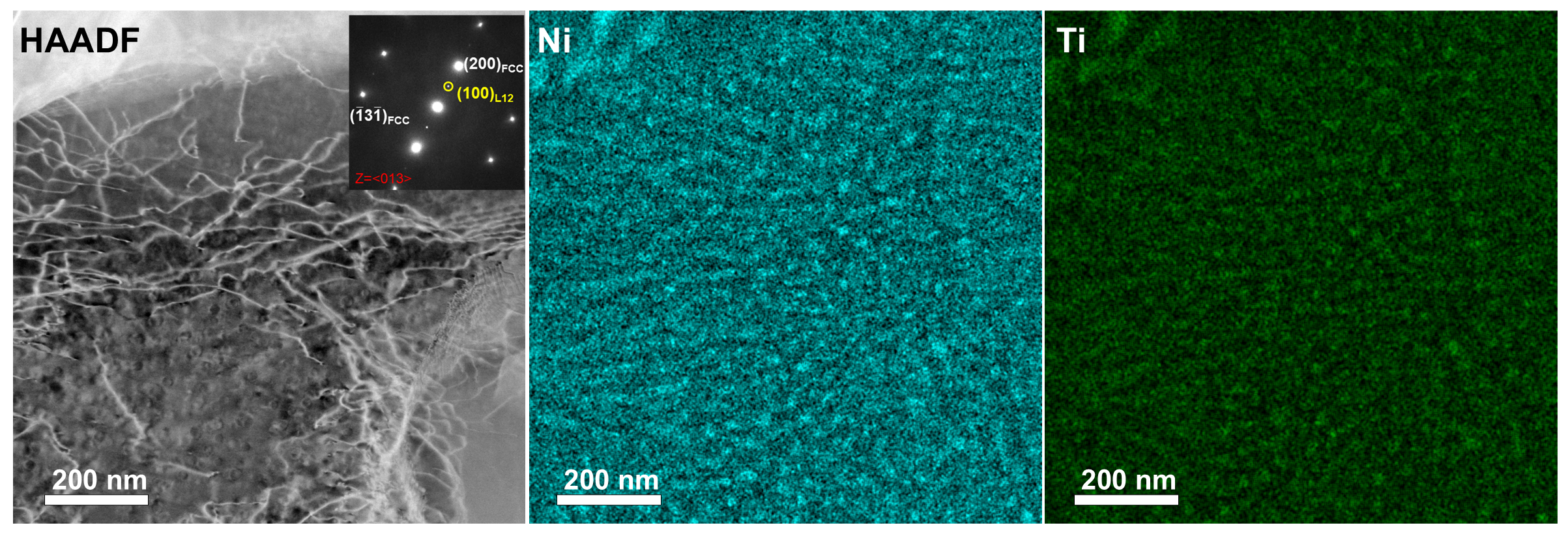


**Figure. S2**. HAADF–STEM image and EDS elemental mapping of a recrystallized grain in the ultrafine-grained region after annealing at 900 °C for 10 min. The corresponding Ni and Ti maps reveals the presence of discontinuous precipitates within the recrystallized grain at this stage.


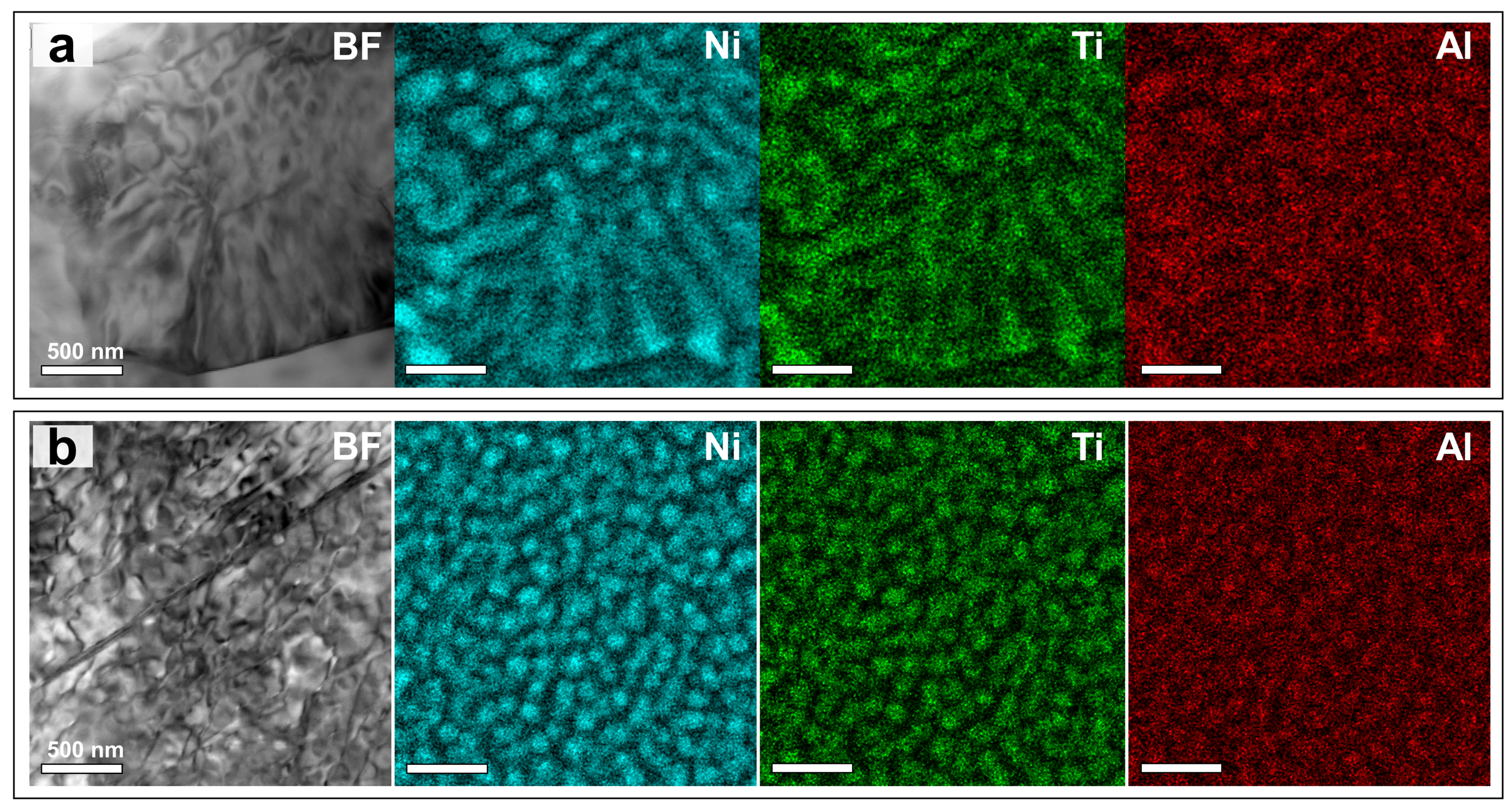


**Figure S3.** Dual-mode precipitation in the BHA alloy. **a.** Continuous precipitates within the non-recrystallized coarse grains. **b.** Discontinuous precipitates inside the recrystallized fine grains, along with the elemental distribution for both types of precipitates.


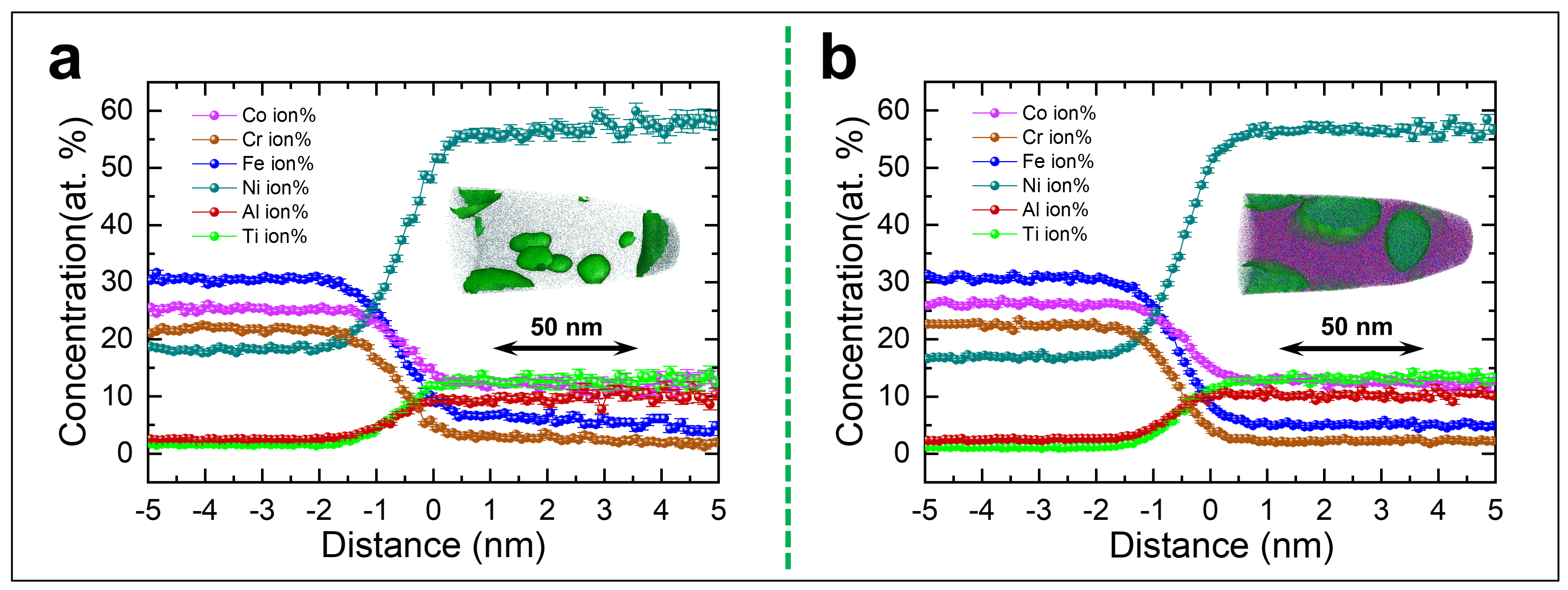


**Figure S4.** APT characterization of elemental partitioning and concentration profiles for (**a**) discontinuous precipitates and (**b**) continuous precipitates.


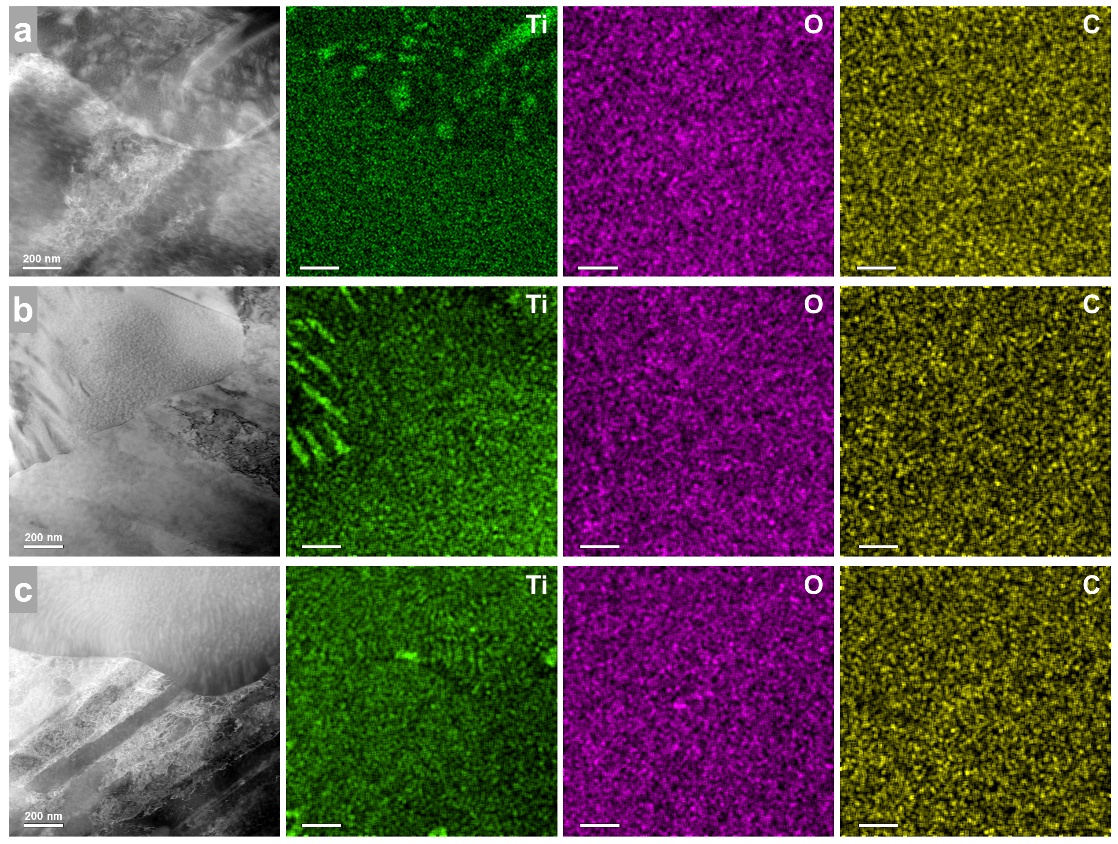


**Figure. S5.** STEM image and corresponding EDS elemental maps acquired from multiple locations near the core–shell interface.


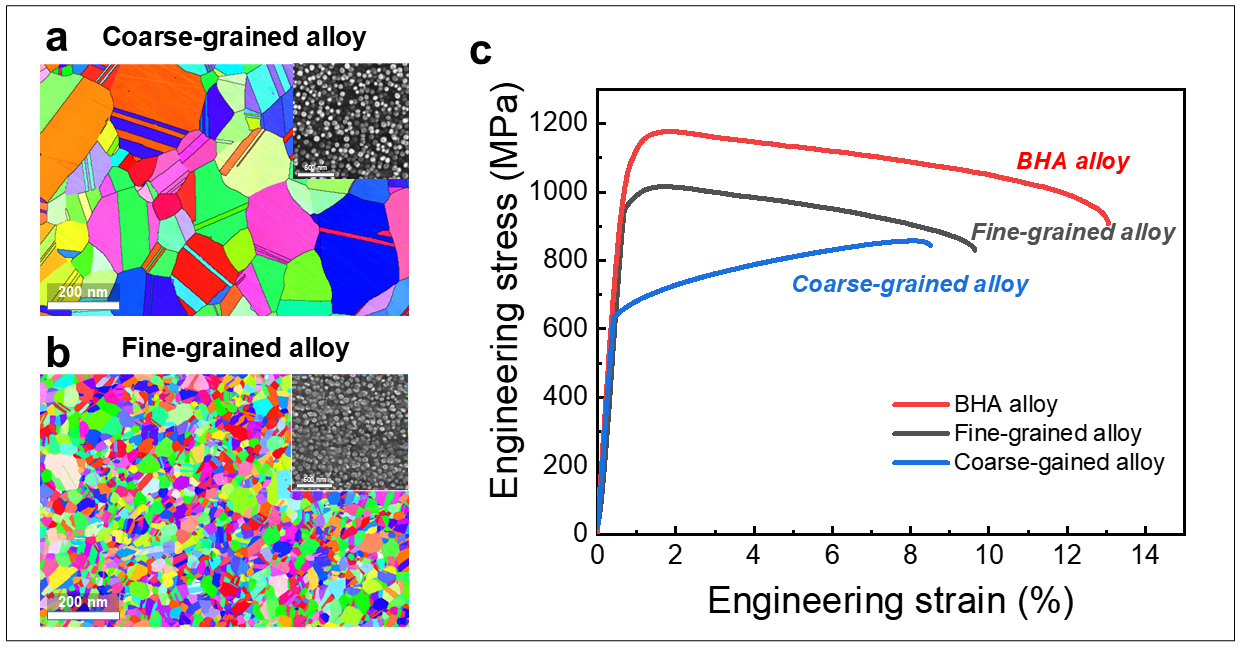


**Figure S6. a.** Microstructure of the coarse-grained sample. **b.** Microstructure of the fine-grained sample. **c.** Engineering stress–strain curves of the coarse-grained, fine-grained, and BHA samples tested at 650 °C.


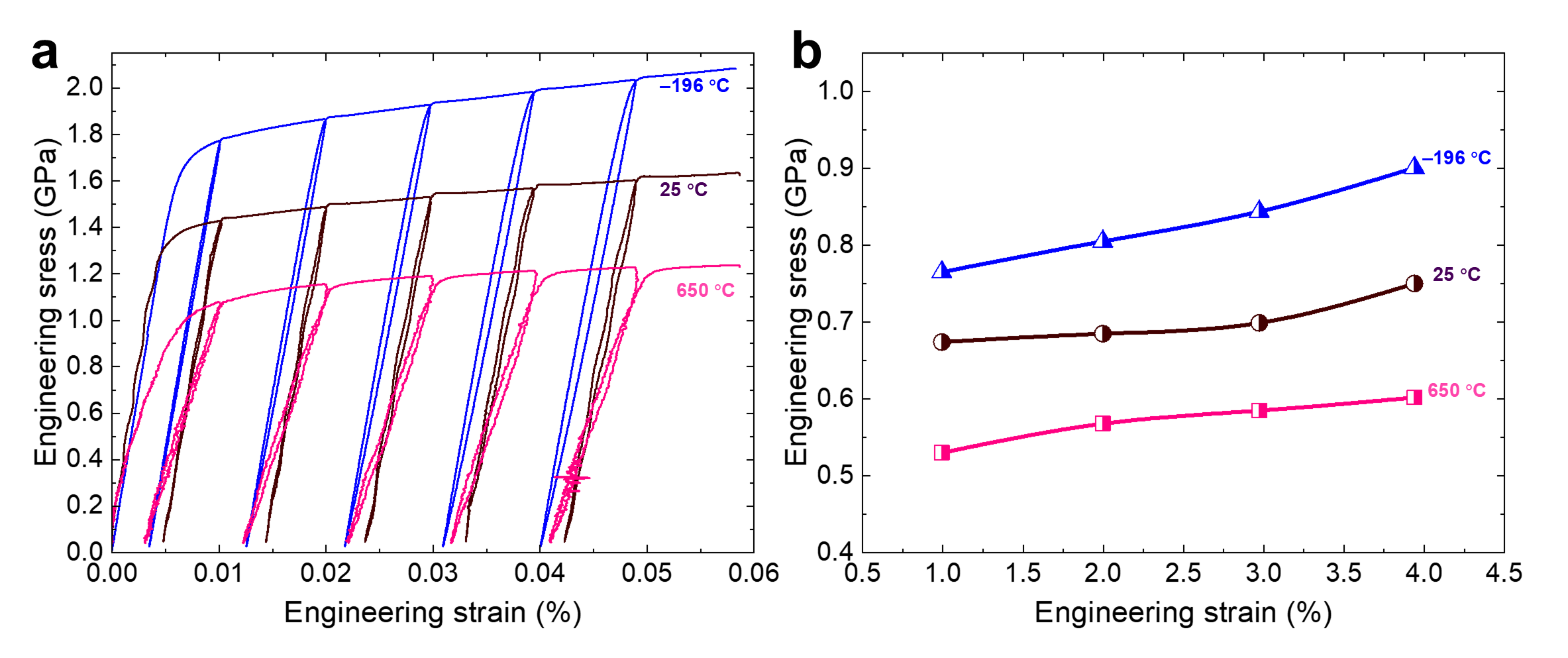


**Figure S7. a.** Loading–unloading–reloading (LUR) curves of the BHA alloy at −196 °C, 25 °C, and 650 °C. **b.** The measured extra stress as a function of applied strain.


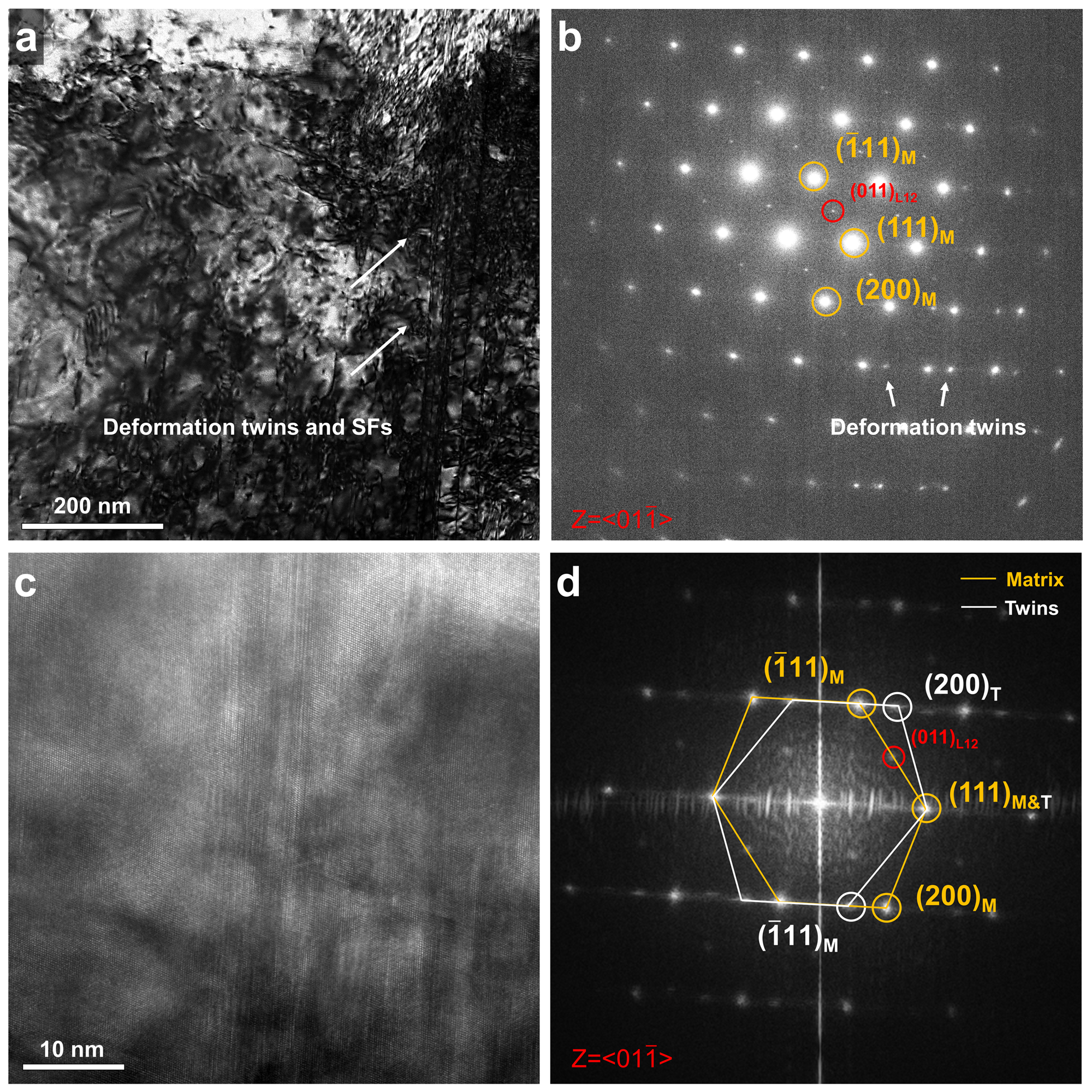


**Figure S8.** **a.** Deformation microstructure in the coarse-grained regions after fracture at ‒196 ℃. **b.** Corresponding SAED pattern confirming the presence of deformation twins. **c.** HR-TEM of the same area. **d.** Corresponding FFT pattern showing additional symmetrical spots and streak-like features, indicative of the formation of deformation twins and stacking faults.


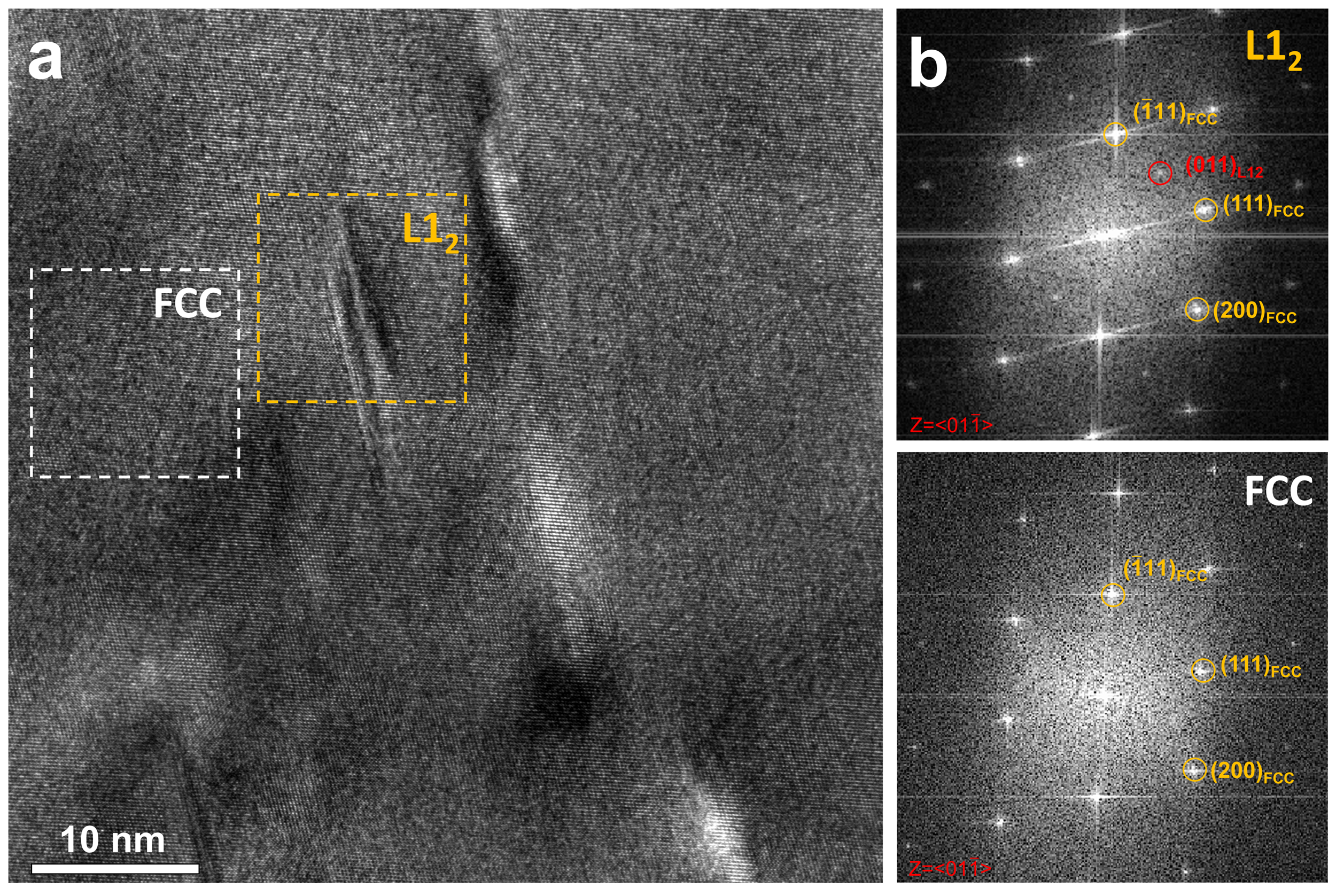


**Figure S9.** HR-TEM image showing the shearing of nano-lamellar precipitates by stacking faults. **b.** FFT patterns corresponding to the boxed regions in **a**, where the presence of streak-like diffraction features indicates the formation of stacking faults.


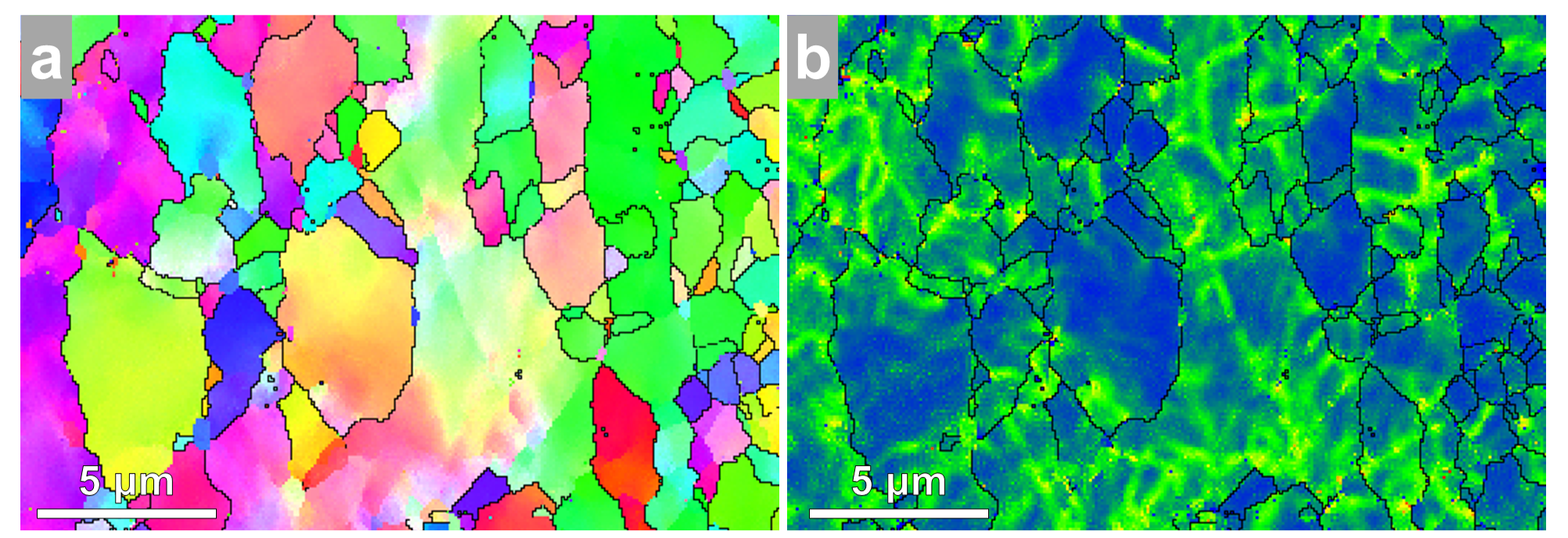


**Figure S10.** **a.** EBSD IPF map and **b.** corresponding GND distribution map of the fine-grained region near the fracture area after tensile deformation at 650 °C.

**
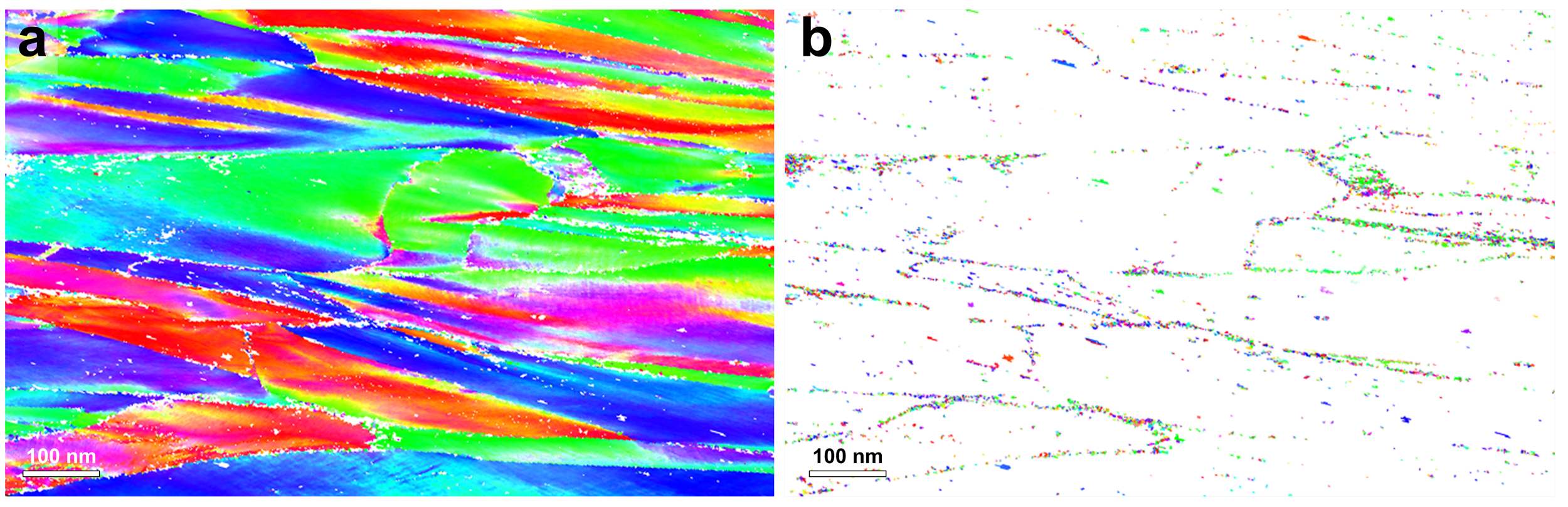
**

**Figure S11.** IPF maps of the subset screened by GOS values. **a.** GOS ≥ 2.5, representing non-recrystallized coarse-grained domains. **b.** GOS < 2.5, representing recrystallized fine-grained domains.

**Table S1.** Compositions of the FCC matrix and nanoprecipitates in the coarse-grained and fine-grained regions.

|  |  | Co (at.%) | Cr  (at.%) | Fe  (at.%) | Ni  (at.%) | Al  (at.%) | Ti  (at.%) |
| --- | --- | --- | --- | --- | --- | --- | --- |
| Coarse-grained | Matrix | 25.88  ±  0.43 | 21.77  ±  0.30 | 29.82  ±  0.33 | 18.13  ±  0.27 | 2.86  ±  0.11 | 1.54  ±  0.08 |
|  | Precipitate | 12.70  ±  0.43 | 2.35  ±  0.19 | 5.27  ±  0.29 | 56.31  ±  0.64 | 10.28  ±  0.39 | 13.09  ±  0.43 |
| Fine-grained | Matrix | 25.37  ±  0.38 | 21.80  ±  0.36 | 30.59  ±  0.40 | 18.14  ±  0.34 | 2.47  ±  0.14 | 1.65  ±  0.11 |
|  | Precipitate | 12.56  ±  0.78 | 1.83  ±  0.36 | 4.51  ±  0.55 | 57.95  ±  1.18 | 10.16  ±  0.71 | 12.98  ±  0.79 |

**Table S2.** Mechanical properties of reference alloys at −196 °C.

| No. | Alloy | YS_MPa | UTS_MPa | Elong_% | YS × EL | Ref. |
| --- | --- | --- | --- | --- | --- | --- |
| 1 | Al6061 | 558 | 711 | 4 | 2288 | ^23^ |
| 2 | Al-Mg-Si alloy | 605 | 705 | 6 | 3812 | ^24^ |
| 3 | 8091-T8X | 574 | 697 | 12 | 6888 | ^25^ |
| 4 | 2195 Al-Cu-Li alloy | 527 | 675 | 16 | 8696 | ^26^ |
| 5 | 2090-T8E41 | 587 | 642 | 14 | 8218 | ^25^ |
| 6 | Al-Cu-Zr alloy | 567 | 627 | 8 | 4763 | ^27^ |
| 7 | 2091-T351 | 442 | 596 | 16 | 7072 | ^25^ |
| 8 | AlCoCrFeNi_2.2_ | 705 | 1151 | 9 | 6557 | ^28^ |
| 9 | AlCoCrFeNi_2.1_ | 857 | 1461 | 16 | 14226 | ^29^ |
| 10 | Fe_35_Mn_15_Cr_15_Ni_25_Al_10_ | 1000 | 1455 | 9 | 9000 | ^30^ |
| 11 | KhN_35_VTYu | 710 | 1200 | 13 | 9017 | ^31^ |
| 12 | Al1.1CoCrFeNi2.1 | 705 | 1151 | 9 | 6557 | ^32^ |
| 13 | Fe_40_Mn_20_Cr_20_Ni_20_ | 1005 | 1140 | 21 | 21105 | ^33^ |
| 14 | Fe_20.4_Mn_20.4_Cr_20.3_Ni_20.3_Co_17.0_N_1.6_ | 613 | 1055 | 11 | 6864 | ^34^ |
| 15 | AlCoCrFeNi_2.1_ | 690 | 1051 | 7 | 4623 | ^28^ |
| 16 | CoCrFeNi | 790 | 1027 | 22 | 18012 | ^35^ |
| 17 | (Fe_40_Mn_40_Co_10_Cr_10_)_96.7_C_3.3_ | 891 | 1025 | 10 | 9088 | ^36^ |
| 18 | CoCrFeNi | 727 | 981 | 16 | 11850 | ^35^ |
| 19 | AlCoCrFeNi_2.0_ | 715 | 952 | 4 | 2645 | ^28^ |
| 20 | Fe_50_Mn_30_Co_10_Cr_10_ | 360 | 920 | 17 | 6120 | ^37^ |
| 21 | Cr_26_Mn_20_Fe_20_Co_20_Ni_14_ | 546 | 830 | 27 | 14742 | ^38^ |
| 22 | CoCrFeNi | 699 | 809 | 8 | 5872 | ^35^ |
| 23 | Nb | 739 | 814 | 28 | 21140 | ^39^ |
| 24 | 316L | 770 | 1113 | 16 | 12320 | ^40^ |
| 25 | Fe-8Mn | 848 | 1083 | 18 | 15264 | ^41^ |
| 26 | Fe_80.4767_C_0.45_Mn_17_Al_2.0_Si_0.05_ | 783 | 1083 | 16 | 12990 | ^42^ |
| 27 | 9% Nickel Steel | 646 | 928 | 10 | 6460 | ^43^ |
| 28 | 7% Ni steel | 744 | 924 | 23 | 17707 | ^44^ |
| 29 | Fe–18Mn–0.6C–1.8Al alloy | 588 | 881 | 25 | 15112 | ^45^ |
| 30 | Ni-20%Fe alloy | 2790 | 2920 | 2 | 5580 | ^46^ |
| 31 | IN718C | 973 | 1146 | 14 | 13136 | ^47^ |
| 32 | Ti-6Al-4V | 1238 | 1354 | 2.9 | 3589 | ^48^ |
| 33 | TA15 | 1018 | 1177 | 13 | 12720 | ^49^ |
| 34 | Ti-6Al-4V ELI | 1000 | 1100 | 19 | 19000 | ^50^ |
| 35 | Ti-3Al-2Zr-1.5Mo alloy | 689 | 1040 | 22 | 14888 | ^51^ |
| 36 | Nb + 5.5% Ti | 724 | 795 | 11 | 8141 | ^39^ |
| 37 | 2195-T8 | 685 | 764 | 12 | 8144 | ^52^ |
| 38 | This work | 1800 | 2100 | 12 | 21600 |  |

**Table S3.** Mechanical properties of reference alloys at 25 °C

| No. | Alloy | YS_MPa | UTS_MPa | Elong_% | YS×EL | Ref. |
| --- | --- | --- | --- | --- | --- | --- |
| 1 | 2195 Al-Cu-Li alloy | 325 | 493 | 18 | 5785 | ^26^ |
| 2 | 8090-T8X | 482 | 534 | 6 | 2892 | ^25^ |
| 3 | 2195 Al-Cu-Li alloy | 499 | 556 | 11 | 5589 | ^26^ |
| 4 | Al-Cu-Zr alloy | 400 | 570 | 5 | 1920 | ^27^ |
| 5 | 8091-T8X | 537 | 581 | 6 | 3222 | ^25^ |
| 6 | Ti-3Al-2Zr-1.5Mo alloy | 501 | 604 | 12 | 6053 | ^51^ |
| 7 | Al6061 | 430 | 620 | 1 | 559 | ^23^ |
| 8 | Al-Mg-Si alloy | 545 | 630 | 2 | 981 | ^24^ |
| 9 | CoCrFeNi | 526 | 740 | 8 | 4366 | ^35^ |
| 10 | CoCrFeNi | 655 | 872 | 16 | 10414 | ^35^ |
| 11 | Al_1.1_CoCrFeNi_2.1_ | 620 | 964 | 16 | 9920 | ^28, 32^ |
| 12 | Al_0.2_Ti_0.3_Co_1.5_CrFeNi_1.5_ | 796 | 989 | 17 | 13532 | ^53^ |
| 13 | Fe_40_Mn_20_Cr_20_Ni_20_ | 925 | 1023 | 7 | 6475 | ^33^ |
| 14 | Fe_35_Mn_15_Cr_15_Ni_25_Al_10_ | 860 | 1260 | 9 | 7740 | ^30^ |
| 15 | Steel 04G2B | 420 | 540 | 31 | 13020 | ^54^ |
| 16 | 316L | 500 | 565 | 18 | 9000 | ^55^ |
| 17 | G20Mn5QT cast steel | 330 | 586 | 27 | 8857 | ^56^ |
| 18 | MS Q355 | 483 | 618 | 16 | 7776 | ^57^ |
| 19 | MS Q460 | 673 | 702 | 16 | 10903 | ^57^ |
| 20 | 316LN | 636 | 947 | 9 | 5915 | ^58^ |
| 21 | 316LN | 809 | 1106 | 4 | 3155 | ^58^ |
| 22 | M963 | 896 | 1030 | 6 | 5018 | ^59^ |
| 23 | K445 superalloy | 990 | 1090 | 6 | 5940 | ^60^ |
| 24 | Ti-26Nb-2Hf | 431 | 497 | 15 | 6508 | ^61^ |
| 25 | Ti-24Nb-4Hf | 376 | 505 | 22 | 8159 | ^61^ |
| 26 | Ti-0.66 at% Oeqv | 395 | 559 | 18 | 7114 | ^62^ |
| 27 | CT20 alloy | 572 | 652 | 15 | 8691 | ^63^ |
| 28 | TA15 | 601 | 659 | 26 | 15450 | ^49^ |
| 29 | Ti-15Mo-2Al | 788 | 827 | 21 | 16548 | ^64^ |
| 30 | TNTZO | 860 | 902 | 14 | 12126 | ^65^ |
| 31 | W-20Cu alloy | 648 | 843 | 5 | 2964 | ^66^ |
| 32 | This work | 1435 | 1600 | 11 | 15785 |  |

**Table S4.** Mechanical properties of reference alloys at 650 °C

| No. | Alloy | YS_MPa | UTS_MPa | Elong_% | YS×EL | Ref. |
| --- | --- | --- | --- | --- | --- | --- |
| 1 | Al1.1CoCrFeNi2.1 | 320 | 455 | 11 | 3520 | ^28^ |
| 2 | Al0.5CoCrCuFeNi | 320 | 330 | 5 | 1600 | ^67^ |
| 3 | Ni-30Co-13Fe-15Cr-6Al-6Ti-0.1B | 753 | 1020 | 9 | 7003 | ^68^ |
| 4 | (Ni2Co2FeCr)92Ti4Al4 | 636 | 783 | 8 | 5279 | ^69^ |
| 5 | P91 | 246 | 306 | 24 | 5904 | ^70^ |
| 6 | S690 | 230 | 290 | 8 | 1840 | ^71^ |
| 7 | K445 | 820 | 1100 | 7 | 5740 | ^60^ |
| 8 | GH3535 | 190 | 520 | 23 | 4370 | ^72^ |
| 9 | GH2984 | 510 | 780 | 20 | 10200 | ^73^ |
| 10 | IN792 | 870 | 1050 | 5 | 4350 | ^74^ |
| 11 | GH4033 | 497 | 663 | 12 | 5914 | ^75^ |
| 12 | UNS N07001 | 586 | 793 | 17 | 10196 | ^76^ |
| 13 | IN 740H | 620 | 865 | 14 | 8680 | ^77^ |
| 14 | M963 | 908 | 1043 | 4 | 3267 | ^59^ |
| 15 | This work | 1075 | 1187 | 12 | 12900 |  |

**Table S5.** Calculated *γ*_APB_(*T*) values at different temperatures.

| Temperature | ‒196 ℃ | 25 ℃ | 650 ℃ |
| --- | --- | --- | --- |
| *γ*_APB_(*T*) (J/m^2^) | 0.279 | 0.268 | 0.234 |

**Table S6**. Theoretical strengthening contributions in the coarse-grained and fine-grained domains at ‒196 ℃, 25 ℃, and 650 ℃.

| Temperature | Region | *σ_0_*  (MPa) | Δ*σ_ss_*  (MPa) | Δ*σ_gb_*  (MPa) | Δ*σ_dis_*  (MPa) | Δ*σ_p_*  (MPa) | *σ_y_*  (MPa) |
| --- | --- | --- | --- | --- | --- | --- | --- |
| ‒196 ℃ | Coarse-grained | 320 | 39 | 103 | 436 | 715 | 1613 |
|  | Fine-grained | 320 | 41 | 538 | 182 | 715 | 1796 |
| 25 ℃ | Coarse-grained | 156 | 36 | 103 | 411 | 687 | 1393 |
|  | Fine-grained | 156 | 39 | 538 | 172 | 687 | 1592 |
| 650 ℃ | Coarse-grained | 14 | 27 | 103 | 309 | 600 | 1050 |
|  | Fine-grained | 14 | 29 | 538 | 129 | 600 | 1310 |

Notes: Δ*σ_ss_*, Δ*σ_gb_*, Δ*σ_dis_* and Δ*σ_p_* are the contributions of solid solution strengthening, grain boundary strengthening, dislocation strengthening, and precipitation strengthening, respectively. *σ_y_* is the overall yield strength.

**References**

1. A. Jagetia*, et al.*, Ordering-mediated local nano-clustering results in unusually large Hall-Petch strengthening coefficients in high entropy alloys. *Mater. Res. Lett.* **9**, 213 (2021).

2. R. L. Fleischer, D. Peckner, The strengthening of metals *J. Frankl. Inst.* **277**, 619 (1964).

3. B. Gwalani*, et al.*, High density of strong yet deformable intermetallic nanorods leads to an excellent room temperature strength-ductility combination in a high entropy alloy. *Acta Mater.* **219**, 117234 (2021).

4. Y. Wang*, et al.*, Probing deformation mechanisms of a FeCoCrNi high-entropy alloy at 293 and 77 K using in situ neutron diffraction. *Acta Mater.* **154**, 79 (2018).

5. N. Ali*, et al.*, Strengthening mechanisms in high entropy alloys: A review. *Mater. Today Commun.* **33**, 104686 (2022).

6. F. He*, et al.*, Design of D022 superlattice with superior strengthening effect in high entropy alloys. *Acta Mater.* **167**, 275 (2019).

7. Z. Wu*, et al.*, Temperature dependence of the mechanical properties of equiatomic solid solution alloys with face-centered cubic crystal structures. *Acta Mater.* **81**, 428 (2014).

8. K. E. Shugaev*, et al.*, The Growth of a Grain during the Annealing of Iron Deformed at 250°C by Shear under Pressure. *Phys. Met. Metallogr.* **123**, 979 (2023).

9. M. Mukherjee, Effect of build geometry and orientation on microstructure and properties of additively manufactured 316L stainless steel by laser metal deposition. *Materialia* **7**, 100359 (2019).

10. S. Yoshida*, et al.*, Effect of elemental combination on friction stress and Hall-Petch relationship in face-centered cubic high / medium entropy alloys. *Acta Mater.* **171**, 201 (2019).

11. M. S. K. K. Y. Nartu*, et al.*, Enhanced tensile yield strength in laser additively manufactured Al0.3CoCrFeNi high entropy alloy. *Materialia* **9**, 100522 (2020).

12. L. P. Kubin, A. Mortensen, Geometrically necessary dislocations and strain-gradient plasticity: a few critical issues. *Scr. Mater.* **48**, 119 (2003).

13. H. Gao*, et al.*, Mechanism-based strain gradient plasticity— I. Theory. *J. Mech. Phys. Solids* **47**, 1239 (1999).

14. L. Fan*, et al.*, Ultrahigh strength and ductility in newly developed materials with coherent nanolamellar architectures. *Nat. Commun.* **11**, 6240 (2020).

15. M. Dodaran*, et al.*, Effect of alloying elements on the γ’ antiphase boundary energy in Ni-base superalloys. *Intermetallics* **117**, 106670 (2020).

16. F. Otto*, et al.*, The influences of temperature and microstructure on the tensile properties of a CoCrFeMnNi high-entropy alloy. *Acta Mater.* **61**, 5743 (2013).

17. M. Naeem*, et al.*, Temperature-dependent hardening contributions in CrFeCoNi high-entropy alloy. *Acta Mater.* **221**, 117371 (2021).

18. G. Laplanche*, et al.*, Elastic moduli and thermal expansion coefficients of medium-entropy subsystems of the CrMnFeCoNi high-entropy alloy. *J. Alloys Compd.* **746**, 244 (2018).

19. W. Li*, et al.*, Modeling the effect of temperature on the yield strength of precipitation strengthening Ni-base superalloys. *Int. J. Plast.* **116**, 143 (2019).

20. G. B. Olson, M. Cohen, A general mechanism of martensitic nucleation: Part I. General concepts and the FCC → HCP transformation. *Metall. Trans. A* **7**, 1897 (1976).

21. S. Curtze*, et al.*, Thermodynamic modeling of the stacking fault energy of austenitic steels. *Acta Mater.* **59**, 1068 (2011).

22. S. Curtze, V. T. Kuokkala, Dependence of tensile deformation behavior of TWIP steels on stacking fault energy, temperature and strain rate. *Acta Mater.* **58**, 5129 (2010).

23. E. Moreno‐Valle*, et al.*, Effect of Grain Refinement on the Mechanical Behaviour of an Al6061 Alloy at Cryogenic Temperatures. *AIP Conf. Proc.* **1353**, 505 (2011).

24. E. C. Moreno-Valle*, et al.*, Effect of the grain refinement via severe plastic deformation on strength properties and deformation behavior of an Al6061 alloy at room and cryogenic temperatures. *Mater. Lett.* **65**, 2917 (2011).

25. K. T. Venkateswara Rao*, et al.*, Cryogenic toughness of commercial aluminum-lithium alloys: Role of delamination toughening. *Metall. Trans. A* **20**, 485 (1989).

26. Y. Peng*, et al.*, Microstructures and cryogenic mechanical properties of spray deposited 2195 Al–Cu–Li alloy with different heat treatments. *Mater. Sci. Eng. A* **895**, 146173 (2024).

27. T. S. Orlova*, et al.*, Influence of decreased temperature on the plasticization effect in high-strength Al-Cu-Zr alloy. *J. Alloys Compd.* **931**, 167540 (2023).

28. Y. Lu*, et al.*, Directly cast bulk eutectic and near-eutectic high entropy alloys with balanced strength and ductility in a wide temperature range. *Acta Mater.* **124**, 143 (2017).

29. T. Bhattacharjee*, et al.*, Effect of low temperature on tensile properties of AlCoCrFeNi2.1 eutectic high entropy alloy. *Mater. Chem. Phys.* **210**, 207 (2018).

30. D. Liu*, et al.*, The cobalt-free Fe35Mn15Cr15Ni25Al10 high-entropy alloy with multiscale particles for excellent strength-ductility synergy. *Intermetallics* **163**, 108064 (2023).

31. P. F. Koshelev*, et al.*, Mechanical properties of alloy KhN35VTYu at low temperatures. *Metal Science and Heat Treatment* **33**, 388 (1991).

32. W. Jiang*, et al.*, Impact of Temperature on the Tensile Properties of Hypereutectic High-Entropy Alloys. *Coatings* **13**, 1836 (2023).

33. Q. X. Ma*, et al.*, High strength and ductility in partially recrystallized Fe40Mn20Cr20Ni20 high-entropy alloys at cryogenic temperature. *Microstructures* **2**, 15 (2022).

34. E. G. Astafurova*, et al.*, Temperature dependence of tensile behavior, deformation mechanisms and fracture in nitrogen-alloyed FeMnCrNiCo(N) Cantor alloys. *J. Alloys Compd.* **925**, 166616 (2022).

35. G. Huang*, et al.*, Nanotwining induced by tensile fatigue and dynamic impact of laser powder bed fusion additively manufactured CoCrFeNi high-entropy alloy. *Journal of Materials Science &amp; Technology* **183**, 241 (2024).

36. L. B. Chen*, et al.*, Ductile-brittle transition of carbon alloyed Fe40Mn40Co10Cr10 high entropy alloys. *Mater. Lett.* **236**, 416 (2019).

37. R. Wei*, et al.*, Toughening FeMn-based high-entropy alloys via retarding phase transformation. *Journal of Materials Science &amp; Technology* **51**, 167 (2020).

38. W. Jiang*, et al.*, Coupling effect of temperature and strain rate on mechanical properties and deformation mechanisms of Cr26Mn20Fe20Co20Ni14 high-entropy alloy. *Mater. Sci. Eng. A* **901**, 146525 (2024).

39. M. I. Bychkova*, et al.*, Effect of titanium on the mechanical properties of niobium at low temperatures. *Metal Science and Heat Treatment* **25**, 707 (1983).

40. P. Mishra*, et al.*, Microstructural Characterization and Mechanical Properties of L-PBF Processed 316 L at Cryogenic Temperature. *Materials* **14**, 5856 (2021).

41. S. K. Hwang, J. W. Morris, The improvement of cryogenic mechanical properties of Fe-12 Mn and Fe-8 Mn alloy steels through thermal/mechanical treatments. *Metall. Trans. A* **10**, 545 (1979).

42. J. Lee*, et al.*, Effects of Mn Addition on Tensile and Charpy Impact Properties in Austenitic Fe-Mn-C-Al-Based Steels for Cryogenic Applications. *Metall. Mater. Trans. A* **45**, 5419 (2014).

43. R. H. van der Jagt, J. Beyer, Thermal and thermomechanical treatment of 9% nickel steel. *Archiv für das Eisenhüttenwesen* **50**, 389 (1979).

44. H.-W. Cao*, et al.*, Effect of Mn Content on Microstructure and Cryogenic Mechanical Properties of a 7% Ni Steel. *Acta Metallurgica Sinica (English Letters)* **31**, 699 (2018).

45. G.-k. Yang*, et al.*, Enhancement of low-temperature toughness of Fe–Mn–C–Al alloy by rare earth Ce-modified inclusions. *Journal of Iron and Steel Research International* **31**, 157 (2023).

46. E. D. Tabachnikova*, et al.*, Mechanical properties of nanocrystalline Ni-20%Fe alloy at temperatures from 300 to 4.2K. *Mater. Sci. Eng. A* **503**, 110 (2009).

47. M. A. Jaswin, D. M. Lal, Effect of cryogenic treatment on the tensile behaviour of En 52 and 21-4N valve steels at room and elevated temperatures. *Mater. Des.* **32**, 2429 (2011).

48. G. Singh*, et al.*, Deformation and strength of Ti–6Al–4V alloyed with B at cryogenic temperatures. *Mater. Sci. Eng. A* **611**, 45 (2014).

49. J. Li*, et al.*, Correction of the constitutive model and analysis of chip formation in cryogenic machining of TA15 titanium alloy. *Journal of Manufacturing Processes* **113**, 16 (2024).

50. E. D. Tabachnikova*, et al.*, Mechanical characteristics, failure regularities, and dimple structures on failure surfaces of Ti–6Al–4V ‘ELI’ ultrafine-grained alloy at temperatures from 300 to 4.2K. *Mater. Sci. Eng. A* **503**, 106 (2009).

51. R. Zhang*, et al.*, New findings on the cryogenic TWIP effect in Ti-3Al-2Zr-1.5Mo alloy with lamellar microstructure. *Scr. Mater.* **229**, 115385 (2023).

52. T. Wang*, et al.*, Influence of Cryogenic Temperatures on the Mechanical Properties and Microstructure of 2195-T8 Alloy. *Metals* **13**, 740 (2023).

53. S. Dasari*, et al.*, Discontinuous precipitation leading to nano-rod intermetallic precipitates in an Al0.2Ti0.3Co1.5CrFeNi1.5 high entropy alloy results in an excellent strength-ductility combination. *Mater. Sci. Eng. A* **805**, 140551 (2021).

54. L. V. Popova*, et al.*, Properties of steel 04G2B with yttrium. *Metal Science and Heat Treatment* **25**, 698 (1983).

55. R. Bidulský*, et al.*, Case Study of the Tensile Fracture Investigation of Additive Manufactured Austenitic Stainless Steels Treated at Cryogenic Conditions. *Materials* **13**, 3328 (2020).

56. Y. Yin*, et al.*, Material parameters in void growth model for G20Mn5QT cast steel at low temperatures. *Construction and Building Materials* **243**, 118123 (2020).

57. J. Xie*, et al.*, Mechanical properties of Q235∼Q460 mild steels at low temperatures. *Construction and Building Materials* **363**, 129850 (2023).

58. S. Wu*, et al.*, Mechanical properties and microstructure evolution of cryogenic pre-strained 316LN stainless steel. *Cryogenics* **121**, 103388 (2022).

59. L. Z. He*, et al.*, Low ductility at intermediate temperature of Ni–base superalloy M963. *Mater. Sci. Eng. A* **380**, 340 (2004).

60. G.-x. Yang*, et al.*, High temperature tensile properties and fracture behavior of cast nickel-base K445 superalloy. *Progress in Natural Science: Materials International* **21**, 418 (2011).

61. R. Yang*, et al.*, Mechanical behaviour of Ti-Nb-Hf alloys. *Mater. Sci. Eng. A* **740-741**, 398 (2019).

62. V. A. Moskalenko*, et al.*, Low temperature peculiarities of plastic deformation in titanium and its alloys. *Cryogenics* **20**, 503 (1980).

63. R. Zhang*, et al.*, Simultaneous improvement in strength and ductility of CT20 titanium alloy at cryogenic temperature. *Materials &amp; Design* **235**, 112416 (2023).

64. M. C. Zang*, et al.*, Cryogenic tensile properties and deformation behavior of a superhigh strength metastable beta titanium alloy Ti–15Mo–2Al. *Mater. Sci. Eng. A* **817**, 141344 (2021).

65. Y. Li*, et al.*, Strength-Ductility Synergy in a Metastable β Titanium Alloy by Stress Induced Interfacial Twin Boundary ω Phase at Cryogenic Temperatures. *Materials* **13**, 4732 (2020).

66. W. Su*, et al.*, Mechanical and thermal properties of W-20Cu alloy for packaging HgCdTe IRFPA detector at cryogenic temperatures. *Cryogenics* **136**, 103758 (2023).

67. C.-W. Tsai*, et al.*, Effect of temperature on mechanical properties of Al0.5CoCrCuFeNi wrought alloy. *J. Alloys Compd.* **490**, 160 (2010).

68. B. X. Cao*, et al.*, Intermediate temperature embrittlement in a precipitation-hardened high-entropy alloy: The role of heterogeneous strain distribution and environmentally assisted intergranular damage. *Mater. Today Phys.* **24**, 100653 (2022).

69. S. Liu*, et al.*, Oxidation behaviors and mechanical properties of L12-strengthened high-entropy alloys at 700℃. *Corros. Sci.* 110499 (2022).

70. X. Guo*, et al.*, Microstructures and high-temperature mechanical properties in 9Cr–0.5Mo–1.8W–VNb steel after aging at 650°C. *Mater. High Temp.* **32**, 566 (2015).

71. C. Maraveas*, et al.*, Mechanical properties of High and Very High Steel at elevated temperatures and after cooling down. *Fire Science Reviews* **6**, (2017).

72. F. F. Han*, et al.*, The tensile behavior of GH3535 superalloy at elevated temperature. *Mater. Chem. Phys.* **182**, 22 (2016).

73. Z. Zhong*, et al.*, Tensile Properties and Deformation Characteristics of a Ni-Fe-Base Superalloy for Steam Boiler Applications. *Metall. Mater. Trans. A* **45**, 343 (2013).

74. B. Du*, et al.*, Investigation on the microstructure and tensile behavior of a Ni-based IN792 superalloy. *Adv. Mech. Eng.* **10**, 1 (2018).

75. J. Ma*, et al.*, Tensile properties and temperature-dependent yield strength prediction of GH4033 wrought superalloy. *Mater. Sci. Eng. A* **676**, 165 (2016).

76. A. K. Roy*, et al.*, Tensile Deformation of a Nickel-base Alloy at Elevated Temperatures. *J. Mater. Eng. Perform.* **17**, 607 (2008).

77. R. K. Singh, J. K. Sahu, Yield strength anomaly and dynamic strain ageing behaviour of recently developed advanced ultra-supercritical boiler grade wrought Ni-based superalloy IN 740H. *Mater. High Temp.* **36**, 220 (2018).
